# Enzymatic Late‐Stage Modifications: Better Late Than Never

**DOI:** 10.1002/anie.202014931

**Published:** 2021-03-08

**Authors:** Elvira Romero, Bethan S. Jones, Bethany N. Hogg, Arnau Rué Casamajo, Martin A. Hayes, Sabine L. Flitsch, Nicholas J. Turner, Christian Schnepel

**Affiliations:** ^1^ School of Chemistry The University of Manchester Manchester Institute of Biotechnology 131 Princess Street Manchester M1 7DN United Kingdom; ^2^ Compound Synthesis and Management Discovery Sciences, BioPharmaceuticals R&D AstraZeneca Gothenburg Sweden

**Keywords:** diversification, enzyme engineering, late-stage functionalisation, oxyfunctionalisation, selectivity

## Abstract

Enzyme catalysis is gaining increasing importance in synthetic chemistry. Nowadays, the growing number of biocatalysts accessible by means of bioinformatics and enzyme engineering opens up an immense variety of selective reactions. Biocatalysis especially provides excellent opportunities for late‐stage modification often superior to conventional de novo synthesis. Enzymes have proven to be useful for direct introduction of functional groups into complex scaffolds, as well as for rapid diversification of compound libraries. Particularly important and highly topical are enzyme‐catalysed oxyfunctionalisations, halogenations, methylations, reductions, and amide bond formations due to the high prevalence of these motifs in pharmaceuticals. This Review gives an overview of the strengths and limitations of enzymatic late‐stage modifications using native and engineered enzymes in synthesis while focusing on important examples in drug development.

## Graphical abstract

Late‐stage modification is an indispensable approach for compound diversification in synthesis. Since enzymes offer particularly powerful means for mild, catalyst‐controlled functionalisations, in recent years several novel biocatalytic methods have emerged enabling a broad array of orthogonal reactions. Herein, the wide range of late‐stage biotransformations is reviewed and various enzyme classes along with topical examples are discussed.

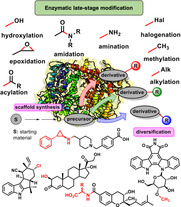

## 1 Introduction

### 1.1 Early‐ vs. Late‐Stage Modification of Multi‐Functional Compounds

C−H functionalisation is fundamental in synthetic chemistry, yet it can be thought of as one of the most challenging reactions. For a long time, the modification of non‐activated carbons in alkanes or arenes is indispensable in providing carbon feedstocks required for synthesis.^[1]^ For example, radical halogenation of aliphatics is a traditional means of functionalising the C−H bond of alkanes. However, the selective C−H modification of more complex molecules is a supreme challenge, since orthogonality and compatibility with existing functionalities are essential preconditions for achieving diversification.

**Scheme 1:**
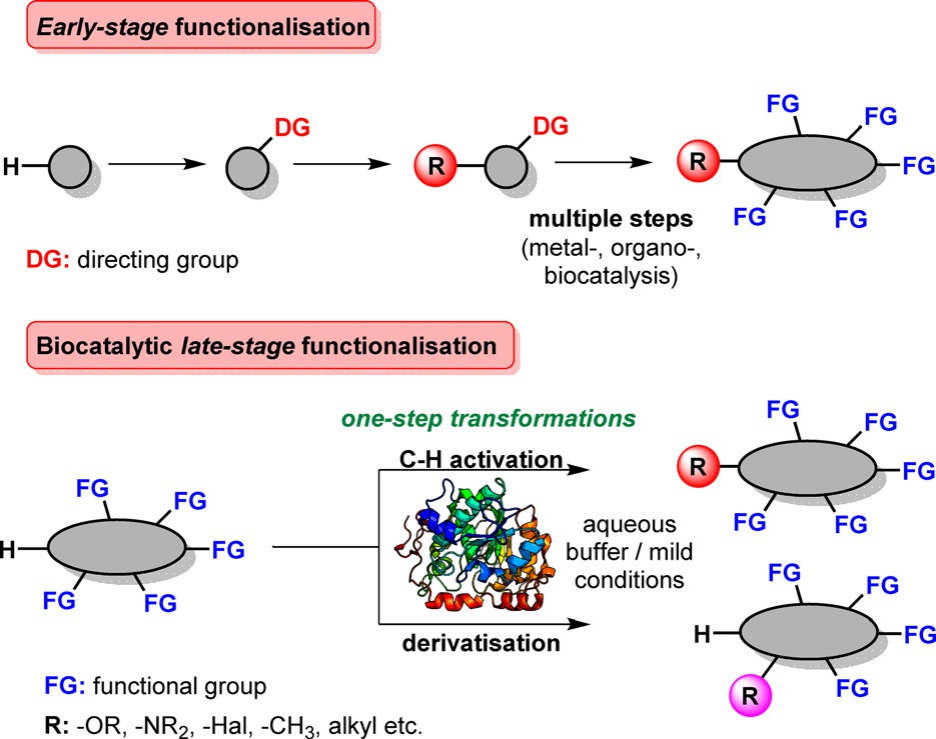
Early‐stage functionalisation generally relies on multiple steps starting from a non‐functionalised starting material. Biocatalytic concepts towards late‐stage modification of various scaffolds allow site‐specific transformations in presence of other functional groups.

Targeted late‐stage modification performed as one of the final synthetic steps as part of a multistep route offers the possibility for diversification, so that C−H and C−heteroatom bonds can be addressed selectively in the presence of other functional moieties (Scheme 1). Among many areas of application, drug discovery and natural product derivatisation profit tremendously from achievements in late‐stage functionalisation (LSF): Compound libraries are typically built up and modified from easily accessible building blocks, paving the way for success in pharmaceutical development. Despite obvious advantages, compatibility and avoidance of cross‐reactivity are major demands that need to be addressed during late‐stage modification.

Metal catalysis is undoubtedly at the forefront of LSFs where C−O, C−N, and C−C bond formation, as well as halogenation are most sought after.^[2]^ In addition organocatalysis, photo‐, and also electrochemistry have proven useful within this field.[^3^, ^4^, ^5^] In recent years, biocatalysis has gratifyingly emerged as a new methodology for LSF. Despite the immense number of highly functionalised molecules in a cell, enzymes enable the synthesis of complex metabolites in aqueous systems, without the need for protecting or directing groups, thus making enzymes ideal catalysts for LSF.

Nowadays, both the bulk and fine chemical industries make use of enzymes in manufacturing processes for achieving selectivity and sustainability. The importance of biocatalytic transformations in industry was recently reviewed in depth by Wu et al.^[6]^ In 2018, a retrosynthetic perspective on how biocatalysis can work hand in hand with chemocatalysis to facilitate synthesis was reported.^[7]^ Tremendous research efforts and an endless number of novel enzymes are beginning to unlock the huge potential for late‐stage modifications. This is of increasing interest both from a drug discovery and high‐throughput experimentation perspective where the availability of orthogonal and robust methods is highly desirable.^[8]^ The advent of late‐stage biotransformations along with their rising importance in synthesis has now motivated us to compile the many and various achievements recently made in this field. This Review presents a streamlined state‐of‐the‐art guide to convey a better understanding of the scope and limitations of late‐stage biocatalysis.

### 1.2 Creation of Biocatalyst Diversity

**Figure 1:**
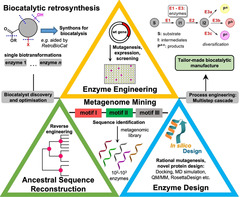
State‐of‐the‐art route towards a tailored bioprocess starting from retrosynthesis up to biocatalytic diversification. The pyramid highlights timely approaches combined to create biocatalyst diversity. To outcompete a conventional synthesis enzymatic late‐stage modification adopts a superior role as it opens access to various derivatives depending on the biocatalyst chosen.

The viability of enzymatic processes in synthetic applications has evolved over the past decades.[^9^, ^10^, ^11^] As in conventional synthesis, the existence of one or multiple (bio)catalysts with optimised reaction conditions is critical in considering enzymes, e.g., for the modification of lead compounds. Biocatalytic retrosynthesis is applied to target molecules to identify plausible transformations and can be supported by recently introduced computer‐aided synthesis planning.^[12]^ Afterwards, enzyme discovery or design and engineering are necessary to create a tailored biocatalytic route (Figure 1).

Nowadays, metagenome mining is useful to discover new biocatalysts from environmental samples. Engineering allows for the optimisation of a biocatalyst towards a desired transformation and to meet the conditions required for a certain process.^[13]^ Undoubtedly, directed evolution, targeted and site‐saturation mutagenesis provide powerful means to diversify and tailor proteins.[^14^, ^15^] Recently, ancestral sequence reconstruction has emerged enabling resurrection of artificial ancestors based on today known variants that are often endowed with higher robustness and a broadened substrate scope.[^16^, ^17^] Further information on generating biocatalyst diversity is found in Supporting Section 1 (see Supporting Information).

### 1.3 C−H Bond Activation: A Background Overview

In general, C−H reactivity is dictated by bond strength, which in turn determines the selectivity of activation. Despite tremendous research on C−H functionalisation, the correlation between bond energetics and reactivity along with the outcome of a reaction is sometimes hard to decipher.^[18]^

**Scheme 2:**
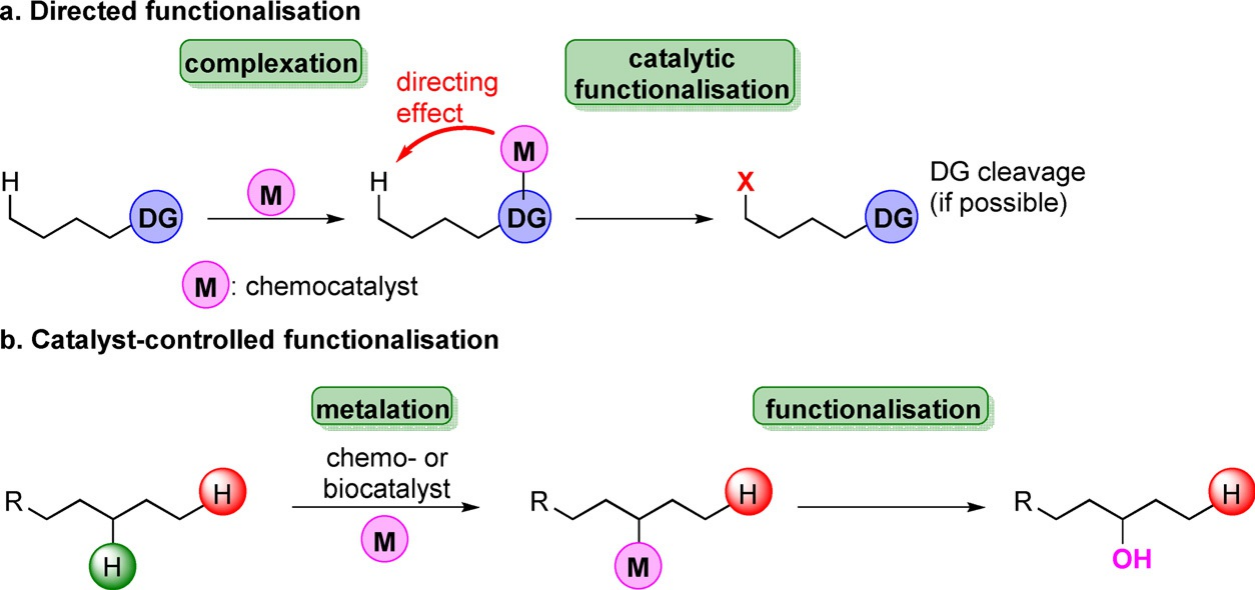
Approaches towards hydrocarbon functionalisation. a) Directed and b) catalyst‐controlled activation are the common methods applied today to address C−H bonds. In (b.) a regioselective hydroxylation is exemplified where a catalyst discriminates between C−H sites.

Not surprisingly, the nature of the C−H bond is determined by its surrounding environment so that electronic, as well as steric properties predominate the ways that bonds are addressed.^[19]^ Table S1 summarises different bond strengths and their impact on reactivity giving insight into how the chemical environment impacts dissociation energy. Directed as well as catalyst‐controlled functionalisation are approaches typically utilised for activation of C−H bonds (Scheme 2).[^20^, ^21^, ^22^] In a directed approach existing functional groups are harnessed to complex a metal ion, for example, while in an ideal catalyst‐controlled reaction merely the (bio)catalyst discriminates between reaction centres permitting site‐specific modification.

## 2 Oxyfunctionalisation: Nature's Manifold Ways of Forming C−O Bonds

**Figure 2:**
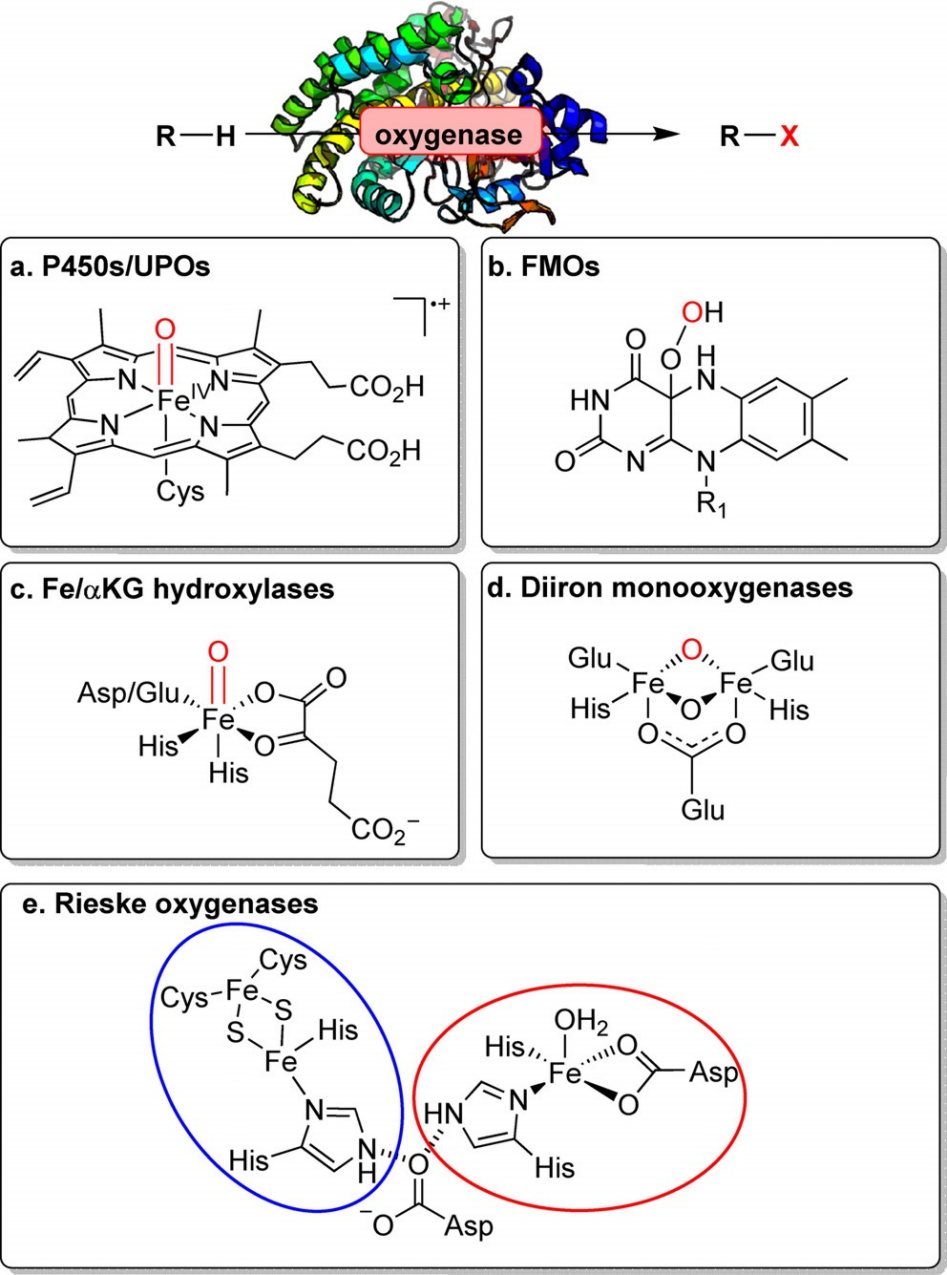
Reactive species facilitating enzymatic oxyfunctionalisation shown for diverse different oxygenase classes. a) P450 monooxygenases and peroxygenases: haem‐thiolate active site showing the active “Compound I” species. b) Flavin‐dependent monooxygenases: flavin hydroperoxide. c) Fe/αKG‐dependent hydroxylases: ferryl‐oxo non‐haem species triggering a radical abstraction from the substrate. d) Diiron monooxygenases: A diiron non‐haem complex of the active site functions as the reactive bridging oxo‐species. e) Rieske oxygenases: non‐haem iron centre (red) bridging to the [2Fe–2S] cluster (blue); adopted from Barry et al.^[31]^

Oxyfunctionalisation within the LSF “toolbox” provides a broad scope consisting of different mono‐, di‐, and peroxygenases able to catalyse the oxidation of non‐activated C−H bonds forming C−O bonds.[^23^, ^24^] Although this transformation is predominantly catalysed by P450s, the breadth of different biocatalysts available for LSF also includes peroxygenases (UPOs),[^25^, ^26^] flavin‐dependent monooxygenases (FMOs),[^27^, ^28^] iron/α‐ketoglutarate‐dependent hydroxylases (Fe/αKGs),^[29]^ diiron monooxygenases,^[30]^ and Rieske oxygenases.[^31^, ^32^] Diversity of these enzymes is emphasised in the variation of their active sites and mechanistic differences (Figure 2). A detailed description of this background can be found in Supporting Section 3 (see Supporting Information). We highlight particularly new and noteworthy advancements of LSF towards different types of C−O bond formation, with particular regard to the implementation of these oxyfunctionalisation reactions in downstream modifications.

### 2.1 Hydroxylation

Natural products are an important source of drug scaffolds due to their structural diversity and biological activity.^[33]^ In many different natural products, drug substrates, and metabolites, endogenous and engineered P450s are prevalent catalysts for in vivo late‐stage C−H oxyfunctionalisation.[^34^, ^35^, ^36^, ^37^]

**Figure 3:**
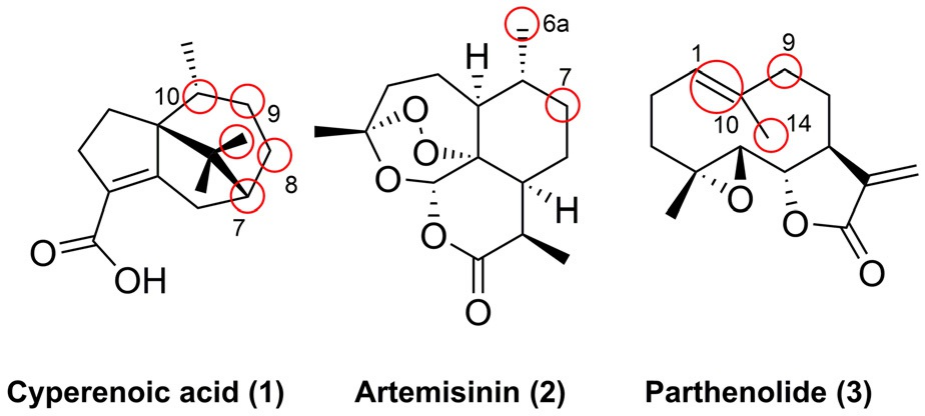
Regio‐ and stereoselective hydroxylation of the sesquiterpene scaffolds. Hydroxylation sites are shown in red.

Hydroxylation often has an essential role for the further modification of natural products, where late‐stage diversification of bioactive compounds is a highly efficient approach for exploring bioactivity and structure–activity relationships (SAR). This has been exemplified for the sesquiterpene class of natural products. In vivo LSF of cyperenoic acid (**1**), an anti‐angiogenic drug, was feasible using the fungus *Cunninghamella elegans* AS 3.2028 that produced different isomers (Figure 3): C*7S*‐, C8*S*‐, C9*R*‐, C10*S*‐, and C11*R*‐hydroxylated products among which the C*7S*‐ and C9*R*‐hydroxy isomers display favourable cytotoxic activity towards two tumour cell lines (HepG2 and MCF‐7).^[38]^ This highlights the relevance of LSF to modulate biological function by introducing a crucial modification. Instead of using an in vivo approach, widely used P450‐BM3 was later rationally engineered to hydroxylate **1**, yielding efficient enzyme variants for bioactive C7‐ and C9*R*‐hydroxylation with excellent selectivity, highlighting the malleability of the P450 for the diversification of a complex sesquiterpene scaffold.^[39]^

**Scheme 3:**
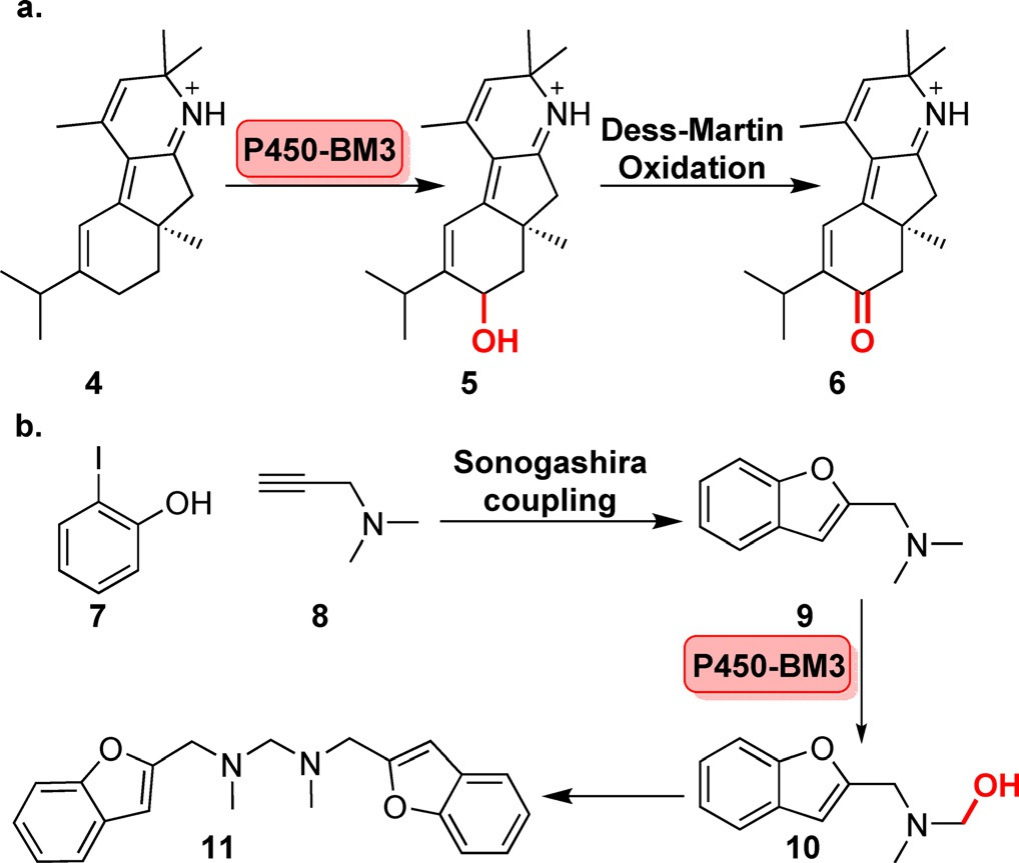
Chemoenzymatic synthesis using engineered P450‐BM3 variants: a) Hydroxylation enables subsequent Dess–Martin oxidation to norditerpenoid alkaloid nigelladine A (**6**); b) alternatively, Sonogashira reaction precedes P450 hydroxylation affording the bis‐2‐substituted benzofuran derivative (**11**).

Engineering of an initially unselective P450‐BM3 variant expanded on a series of different late‐stage hydroxylation products of the antimalarial sesquiterpene lactone, artemisinin (**2**). Three variants displayed a high degree of turnover towards the regio‐ and stereoselective hydroxylation of three C(sp^3^)−H sites providing a chemoenzymatic platform towards novel antimalarial agents.^[40]^ Similarly, late‐stage diversification of the anticancer sesquiterpene lactone parthenolide (**3**) was accomplished from the engineering of the same P450‐BM3 variant, to oxidise two C(sp^3^)−H sites (C9 and C14) and the C1,C10‐double bond.^[41]^ Three enzymes were evolved to catalyse the epoxidation and hydroxylation of **3**, providing important scaffolds for the synthesis of further bioactive derivatives.^[41]^ Furthermore, in the total synthesis of the norditerpenoid alkaloid nigelladine A (**6**), an engineered P450‐BM3 variant was able to catalyse a regioselective allylic oxidation at C7, enabling a subsequent Dess–Martin oxidation to the desired target molecule (Scheme 3). This underscores the advantage of biocatalysis since the use of an array of traditional chemical oxidants resulted in poor selectivity and over‐oxidation.^[42]^

**Figure 4:**
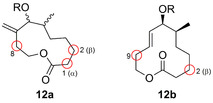
Regioselective hydroxylation of pikromycin motifs **12 a** and **12 b**. Diversity sites are highlighted by a red circle.

Chemoenzymatic synthesis has also been demonstrated in a one‐pot, two‐step cascade, where the first step was a palladium‐free Sonogashira cross‐coupling to a benzofuran (**9**), followed by hydroxylation using a BM3 variant facilitating the loss of formaldehyde and affording the bis‐2‐substituted product (**11**).^[43]^ Furthermore, the chemoenzymatic regio‐ and stereochemical diversification of the macrocyclic skeleton of pikromycin (**12 a**/**b**) was initiated via a combination of click chemistry and esterification prior to hydroxylation catalysed by the engineered P450 PikC (Figure 4).^[44]^ This study emphasised how a P450 triple mutant derived from a biosynthetic pathway could be evolved into a synthetically viable biocatalyst permitting late‐stage diversification of cyclic motifs.

**Figure 5:**
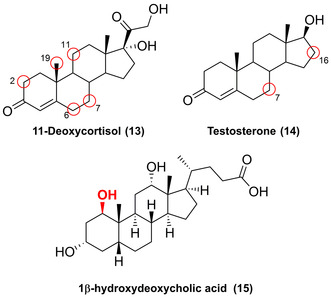
Regio‐ and stereoselective hydroxylation of different steroids. Diversity sites highlighted in the red circle.

The regio‐ and stereoselective late‐stage hydroxylation of steroids proves challenging and often requires multi‐step syntheses, yet selectively decorating the steroid scaffold is essential for drug synthesis.^[45]^ P450s have the capability to overcome this problem: Recently, different wild‐type fungal P450s (STH10, CYP5150AP2, CYP5150AP3, and CYP5150AN1) were shown to have distinct regioselectivities towards the hydroxylation of 11‐deoxycortisol (**13**), yielding 19‐, 11β‐, 7β‐, 6β‐, and 2β‐hydroxy‐11‐deoxycortisol (Figure 5).[^46^, ^47^] The access to the 19‐hydroxylation is pivotal to the chemoenzymatic production of 19‐norsteroidal pharmaceuticals whilst steroidal 7β‐alcohols exhibit anti‐inflammatory and neuroprotective properties.[^46^, ^48^, ^49^]

Thus very recently, P450‐BM3 was engineered to efficiently catalyse the LSF of six different steroids with high regio‐ and stereoselectivity (**14**; adrenosterone; nandrolone; epistosterone; androstenedione; d‐ethylgonenedione) yielding their respective 7β‐alcohols whilst the C16 position with distinctive α‐ and β‐diastereoselectivity succeeded by directed evolution as exemplified for another set of steroids.[^50^, ^51^] Furthermore, a P450‐BM3 mutant was used to selectively synthesise the steroid 1β‐hydroxydeoxycholic acid (**15**) and its deuterated analogue on a milligram scale.^[52]^ Notably, the screening of P450‐BM3 libraries has been shown to be a lucrative endeavour for LSF, with variants oxidising an array of human drugs: chlorzoxazone, testosterone (**14**), amitriptyline, lidocaine, diclofenac, naproxen, and noscapine, thus emphasising the impact of P450 hydroxylations on the synthesis of putative human drug metabolites.[^53^, ^54^]

**Figure 6:**
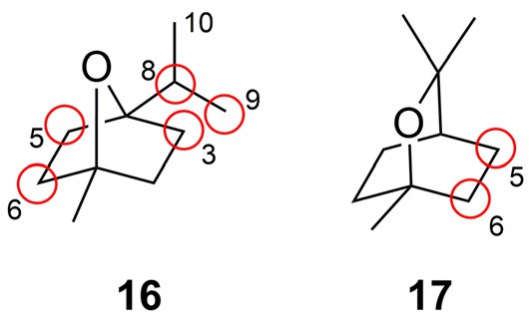
Regioselective hydroxylation of achiral terpenes: 1,4‐cineole (**16**) and 1,8‐cineole (**17**). Diversity sites highlighted by the red circle.

Development of P450s for the production of valuable oxygenated terpene intermediates for further functionalisation also provides a gateway to high value carotenoids and vitamins.^[55]^ For example the engineering of P450cam and P450‐BM3 facilitated a shift in the selective hydroxylation of the monoterpenes 1,4‐ (**16**) and 1,8‐cineole (**17**) inducing two stereocentres (Figure 6).^[56]^

**Figure 7:**
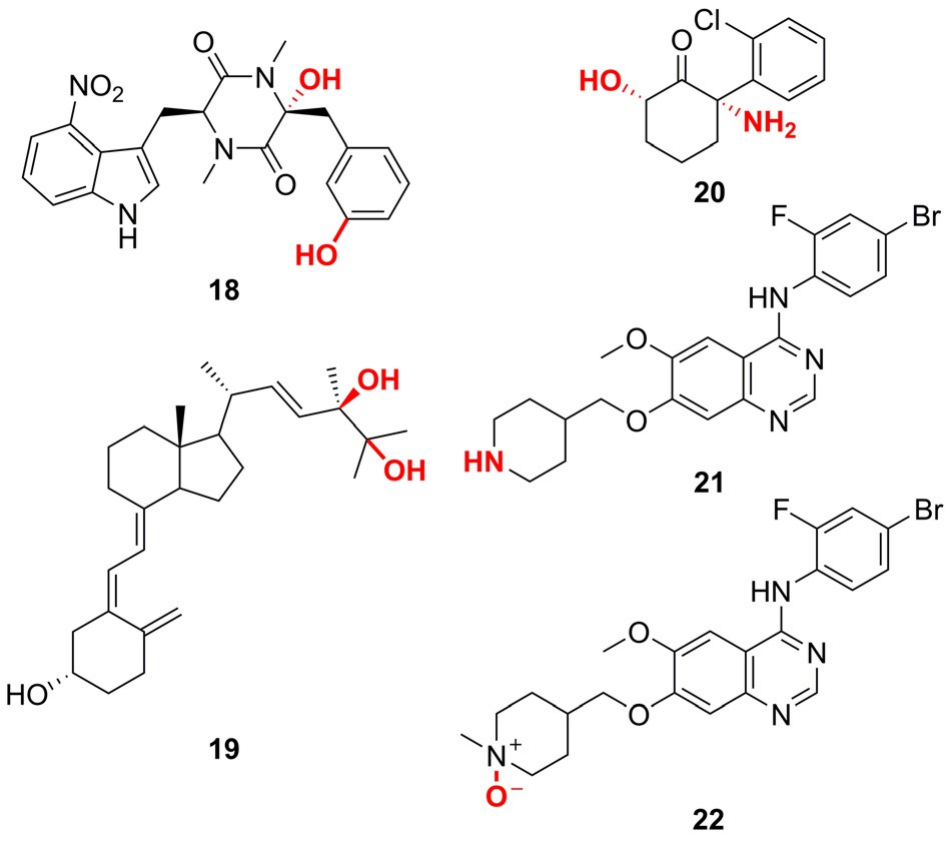
Multi‐oxyfunctionalisation LSF. The aromatic and aliphatic P450 TxtC‐catalysed hydroxylation gives diketopiperazine derivative **18**, whilst dihydroxylation of vitamin D2 catalysed by CYP109E1 gives **19**. The consecutive oxidative N‐demethylation and C6‐hydroxylation of (*S*)‐ketamine towards **20** is catalysed by CYP154E1. Vandetanib oxidation: A P450 catalyses demethylation via oxidation affording *N*‐desmethylvandetanib (**21**) and an FMO‐catalysed oxidation yields vandetanib *N*‐oxide (**22**).

Multi‐oxyfunctionalisation provides an additional approach exploited in biocatalysis. A wild‐type P450, TxtC, is capable of both aliphatic and aromatic C−H hydroxylation in the LSF of a diketopiperazine (**18**).[^57^, ^58^] Moreover, recent examples report on the dihydroxylation of either two aliphatic or aromatic C−H bonds within a vitamin D2 motif and benzene derivatives, respectively.[^59^, ^60^] Wild‐type CYP109E1 has been identified to highly regio‐ and stereoselectively hydroxylate vitamin D2 via two‐step dihydroxylation (Figure 7).^[59]^

Additionally, P450s have been engineered towards multiple oxyfunctionalisations.[^60^, ^61^] A triple mutant of CYP154E1 was able to catalyse the consecutive oxidative N‐demethylation and regio‐and stereoselective C6‐hydroxylation to afford the antidepressant (2*S*,6*S*)‐hydroxynorketamine (**20**) from (*S*)‐ketamine.^[61]^ Furthermore, the use of multiple enzymes for different oxyfunctionalisations has been shown for the anti‐cancer kinase inhibitor, vandetanib, which was oxidised to *N*‐desmethylvandetanib (**21**) and vandetanib *N*‐oxide (**22**) via P450 and FMO, respectively.^[62]^

**Figure 8:**
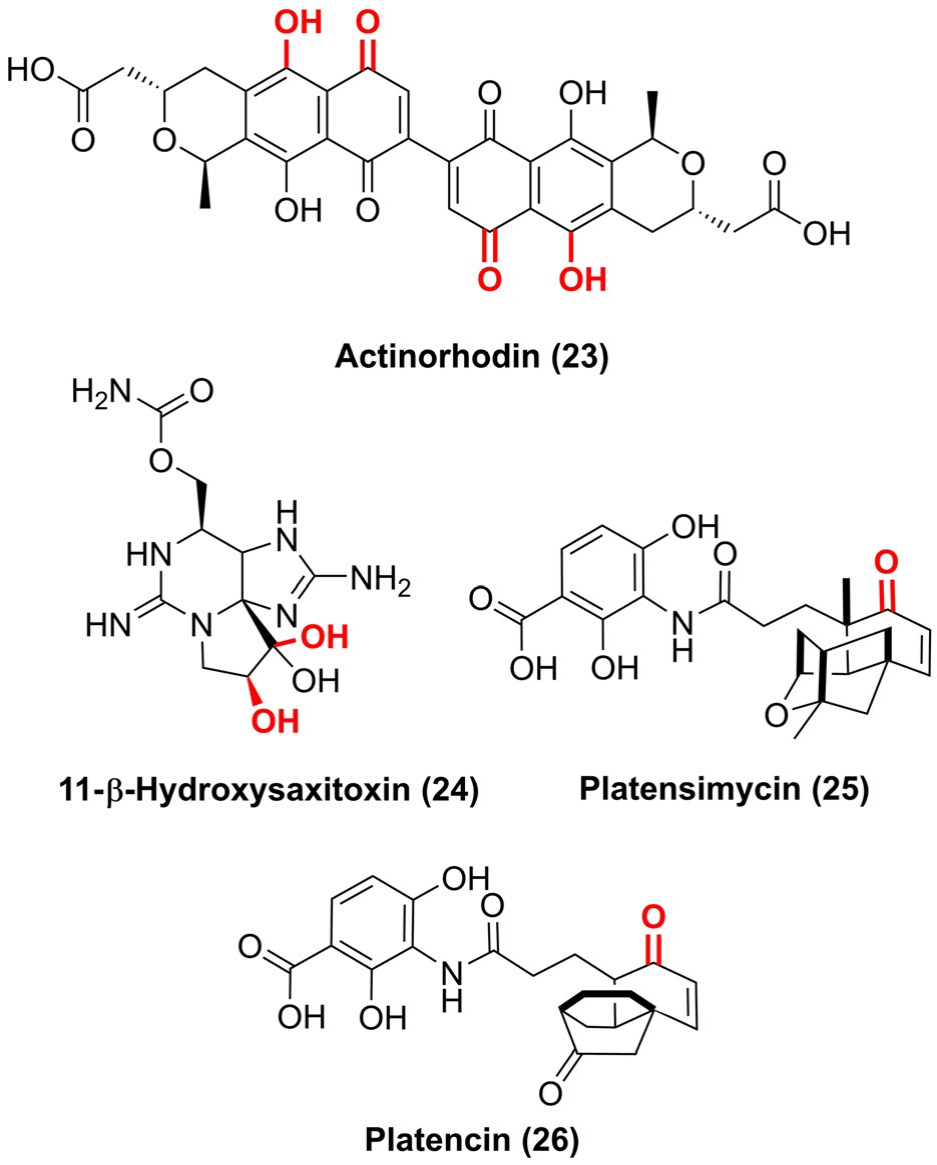
Non‐haem oxygenase‐catalysed hydroxylations within biosynthetic natural product pathways: FMO was shown to catalyse two hydroxylations in the pyranonaphthoquinone antibiotic actinorhodin (**23**) biosynthesis prior to dimerisation. Two Rieske oxygenases introduce the LSF of β‐saxitoxinol to 11‐β‐hydroxysaxitoxin (**24**). A non‐haem diiron monooxygenase hydroxylates platensimycin (**25**) and platencin (**26**) before further oxidation.

LSF hydroxylation reactions catalysed via non‐haem oxygenases from biosynthetic pathways were shown for FMOs, Rieske oxygenases, diiron oxygenases, and Fe/αKG‐dependent ones. FMO‐catalysed chemoenzymatic synthesis yielded a diverse array of stereodivergent azaphilone natural products.^[63]^ Within the biosynthesis of the polyaromatic pyranonaphthoquinone antibiotic actinorhodin (**23**), two consecutive hydroxylations take place at the C6 and C8 positions via a two‐component FMO (Figure 8).^[64]^ Sequential dihydroxylation within the natural product pathway of saxitoxin has recently been shown to be catalysed by two Rieske oxygenases. Each enzyme was responsible for a regio‐ and stereoselective hydroxylation from β‐saxitoxinol to yield 11‐β‐hydroxysaxitoxin (**24**).^[65]^ Furthermore in the biosyntheses of platensimycin (**25**) and platencin (**26**), a non‐haem diiron monooxygenase hydroxylates at the C5 β‐position facilitating facile diversification of these natural products.^[66]^ The breadth of different oxygenases and compound classes available for hydroxylation highlights the essential role of this late‐stage oxyfunctionalisation in Nature.

### 2.2 Epoxidation

**Scheme 4:**
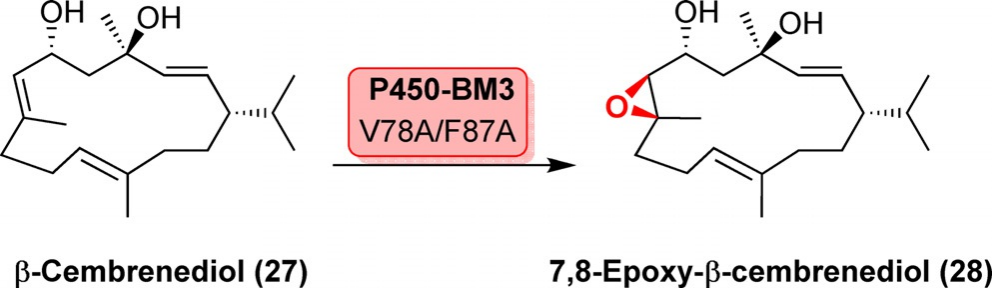
Regio‐ and stereoselective epoxidation of the 14‐membered macrocyclic β‐cembrenediol (**27**) to 7,8‐epoxy‐β‐cembrenediol (**28**).

Late‐stage oxyfunctionalisation of carbon−carbon double bonds into epoxides affords an important motif in many natural products, yet chemoselectivity of epoxidation over an alternative hydroxylation site can be challenging. Notably, late‐stage selective epoxidation of the terpenoid β‐cembrenediol (**27**) exhibited regio‐, chemo‐, and stereoselectivity using P450‐BM3 variants (Scheme 4).^[67]^ Within this 14‐membered macrocycle, three potential epoxidation sites are present, so that control of regioselectivity can pose a challenge.^[67]^ P450‐BM3 V78A/F87A was a parent variant later employed in a rational engineering approach using binding density surface maps to identify residues that could (de‐)stabilise different binding modes, favouring C7,C8‐epoxidation over alternative hydroxylation sites and vice versa.^[68]^ Systematic substrate engineering using different 14‐membered cembranoids resulted in a better understanding of regioselective oxidation by P450‐BM3. The survey revealed that the impact of ring rigidity along with directing groups can strongly impact regioselectivity. In addition, active‐site engineering proved successful for tuning regioselective cembranoid oxidation.^[69]^

**Figure 9:**
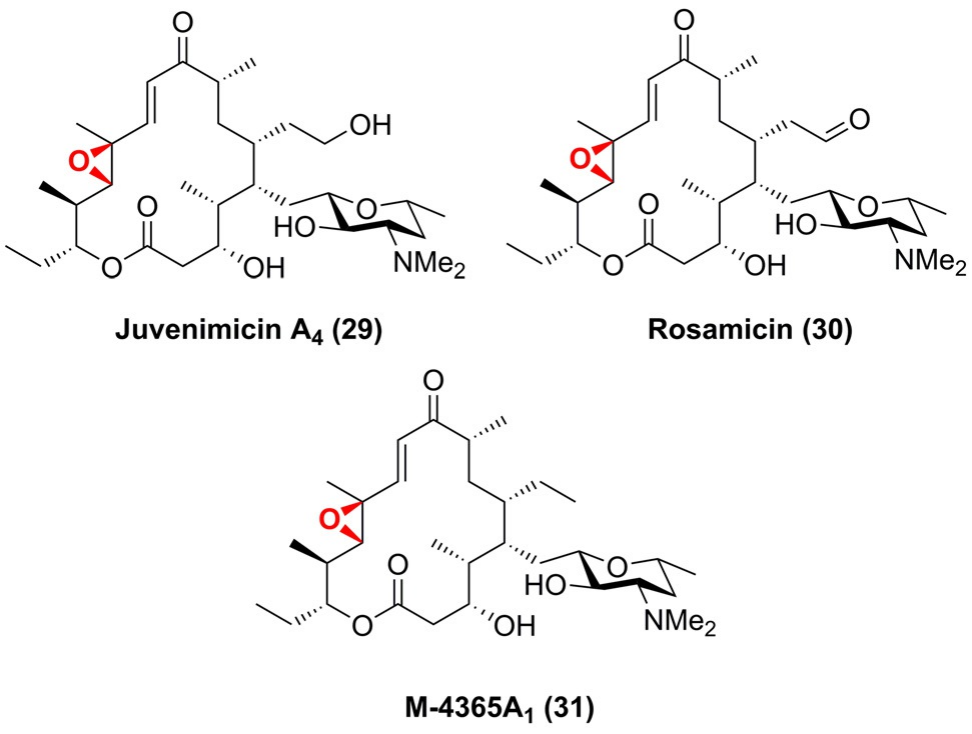
Epoxidation of tylactone‐based antibiotic macrolides: juvenimicin (**29**), rosamicin (**30**), and M‐4365 (**31**) catalysed via a P450 (JuvD).

By combining engineering and in vivo synthesis, the late‐stage C12,C13‐epoxidation into the tylactone‐related antibiotic macrolides juvenimicin (**29**), rosamicin (**30**), and M‐4365 (**31**) became feasible by introducing an artificial chimeric construct consisting of JuvD and reductase P450‐RhF into the biosynthetic pathways (Figure 9).^[70]^

**Scheme 5:**
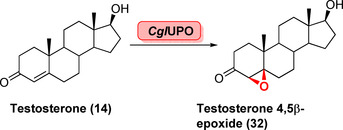
Stereoselective UPO epoxidation of the steroid testosterone (**14**).

In addition to P450s, UPOs and FMOs are important biocatalysts for epoxidation reactions. Recently, a UPO (*Cgl*UPO) has been identified which catalyses the formation of the 4,5β‐epoxide (**32**) from **14** (Scheme 5).^[71]^

*Cgl*UPO displayed high chemo‐ and stereoselectivity and favoured the epoxidation reaction over 16α‐hydroxylation. UPOs provide a suitable alternative to more established P450s; however low tolerance of the co‐substrate H_2_O_2_ is a notable bottleneck, making elaborate in situ regeneration systems necessary. In contrast to P450s, UPOs are underexplored in LSF of complex molecules.

### 2.3 Spirocyclisation

**Figure 10:**
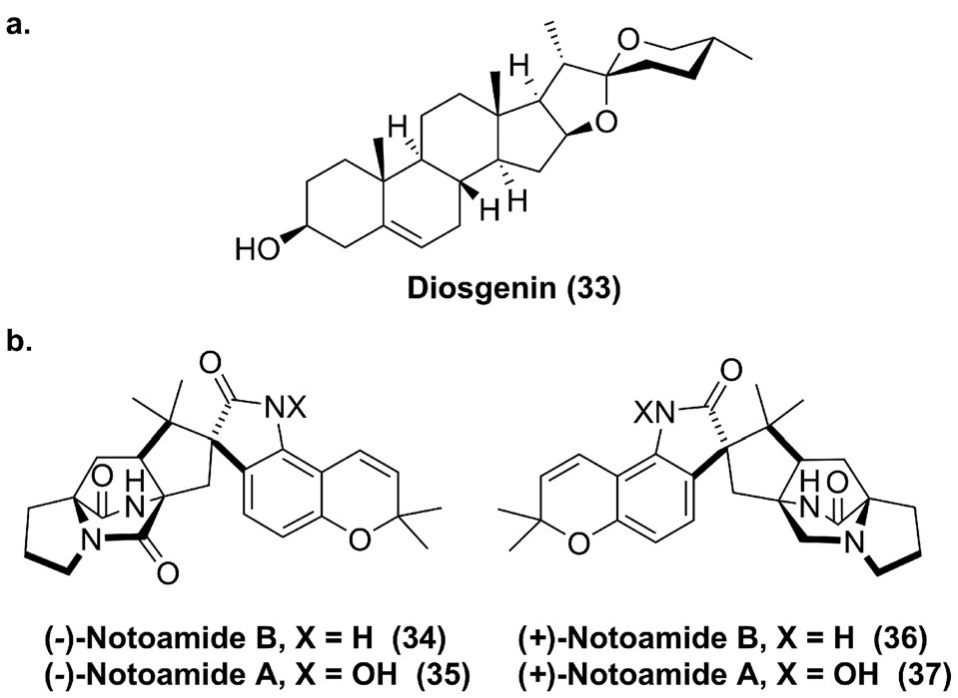
Spiroketal natural products, diosgensin (**33**) and notoamide derivatives (**34**–**37**), where spirocyclisation was afforded by oxyfunctionalisation catalysed by P450s and FMOs, respectively.

The spiro motif is attractive in drug discovery due to its rigidified conformation which can be of importance to favour ligand–receptor interactions.^[72]^ The prominence of spirocyclic compounds within natural product pathways was highlighted by Tang et al. in a review published in 2017.^[24]^ Noteworthy, biocatalytic spirocyclisation is currently limited to a few extraordinary examples originating from natural product biosyntheses as discussed below. Late‐stage dihydroxylation of cholesterol to the 5,6‐furoketal of cholesterol was catalysed via two P450s (*Pp*CYP90G4 and *Tf*CYP90B50), allowing further oxidative cyclisation that produces the spiroketal diosgenin (**33**, Figure 10 a).^[73]^

**Scheme 6:**
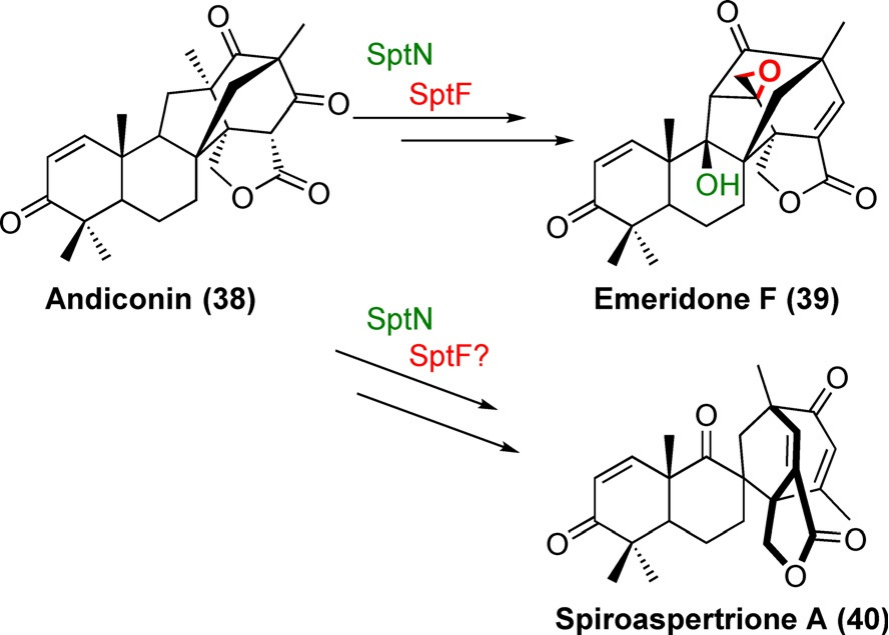
Fe/αKG‐catalysed hydroxylation and epoxidation reactions within the biosynthetic pathway of emeridone F (**39**) from andiconin (**38**) and their predicted involvement within the pathway of the spiro‐meroterpenoids, spiroaspertrione (**40**).

Moreover, the anticancer agents (+)/(−)‐notoamide A/B (**34**–**37**) contain a spiro‐oxindole moiety, introduced biocatalytically using two fungal FMOs (NotI and NotI′) via sequential stereoselective epoxidation and semi‐Pinacol rearrangement (Figure 10 b).^[74]^ Two Fe/αKGs (SptF and SptN) have recently been identified within the gene cluster of the fungal meroterpenoid emeridone F from *Aspergillus* sp. TJ23.^[75]^ These two dioxygenases displayed different oxyfunctionalisations, where SptF catalyses an oxidative rearrangement preceding an epoxidation, while SptN catalyses regio‐ and stereoselective hydroxylation at the C9 position within the core motif of emeridone F (**39**, Scheme 6). Presumably these enzymes are part of the biosynthetic pathway of a spirocyclic emeridone analogue, spiroaspertrione A (**40**).^[75]^ Interestingly, SptF and SptN displayed in vitro activity towards different emeridone derivatives, demonstrating the first potential use in LSF.^[75]^

### 2.4 Deoxyfluorination

Oxyfunctionalisation can also facilitate the incorporation of fluorine as a late‐stage modification via deoxyfluorination. The direct incorporation of fluorine as part of a biohalogenation reaction is discussed in more detail in Section 3.4.

**Figure 11:**
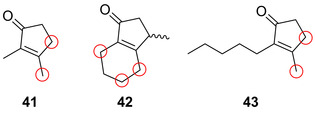
Regioselective chemoenzymatic fluorination of cyclopentenone derivatives **41**–**43**, catalysed via the initial P450‐BM3 variants hydroxylation preceding deoxyfluorination. Diversification sites highlighted in the red circle.

**Scheme 7:**
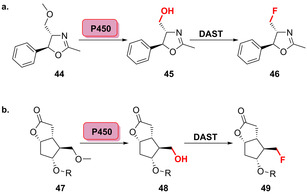
Regioselective chemoenzymatic transformation of a methoxy group into a fluorine substituent of a) 5‐phenyloxazoline derivative **44** and b) Corey lactone derivatives (**47**).

Notably, the biocatalytic regio‐ and stereoselective hydroxylation of drug moieties can be further modified by a chemical fluorination step that was comprehensively exemplified by Rentmeister et al.^[76]^ For the selective two‐step chemo‐enzymatic fluorination of small organic molecules, an engineered P450‐BM3 perfoms a highly regioselective hydroxylation. Cyclopentenones (**41**–**43**) were hydroxylated at 2–3 different sites (Figure 11). Upon purification of the hydroxylated products, deoxyfluorination was carried out using diethylaminosulfur trifluoride (DAST, Scheme 7 a). Moreover, it was shown that the late‐stage transformation could convert a methoxy group into a fluorine substituent. Deoxyfluorination was preceded by hydroxylation and decomposition of the intermediary hemiacetal into the hydroxylated intermediate (Scheme 7 b).

**Scheme 8:**
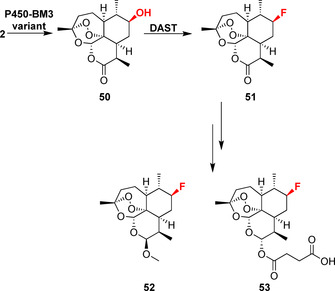
Chemoenzymatic deoxyfluorination of artemisinin (**2**) enabling the further chemical reduction to (7*R*)‐fluoroartemether (**52**) as well as (7*R*)‐fluoroartesunate (**53**).

Additionally, deoxyfluorination of larger terpenoid moieties has been shown via the chemoenzymatic synthesis of the sesquiterpene lactone derivatives (7*R*)‐fluoroartemether (**52**) and (7*R*)‐fluoroartersunate (**53**).^[40]^ The aforementioned engineered P450‐BM3 variant was used to accomplish regio‐ and stereoselective hydroxylation of artemisinin (**2**), followed by deoxyfluorination (Scheme 8). The application of biocatalytically driven deoxyfluorination expands the potential of oxidative enzymes in the late‐stage synthesis of fluorinated drug derivatives. P450s achieve the stereoselective heterofunctionalisation of complex molecular scaffolds, thus providing a useful alternative to much rarer fluorinases (Section 3.4).

### 2.5 Non‐Native Oxyfunctionalisation

Non‐native oxyfunctionalisation is an emerging field that facilitates the exploitation of engineered biocatalysts towards non‐natural reactions: Carbene transfer,[^77^, ^78^, ^79^, ^80^, ^81^, ^82^, ^83^] C−H amination,[^84^, ^85^, ^86^, ^87^] S−N sulfimidation,^[88]^ Si−H hydroxylation,^[89]^ and aziridination provide topical examples.^[90]^ These novel functionalisations have been accentuated in reviews on applications and engineering that are dominated by P450s.[^91^, ^92^, ^93^] Common in many of these non‐natural activities is the mutation of the conserved proximal Cys to a Ser residue within P450‐BM3, which raised the reduction potential of the ferric state; these novel enzymes were named P411s due to the change of characteristic spectroscopic properties.^[78]^

**Scheme 9:**
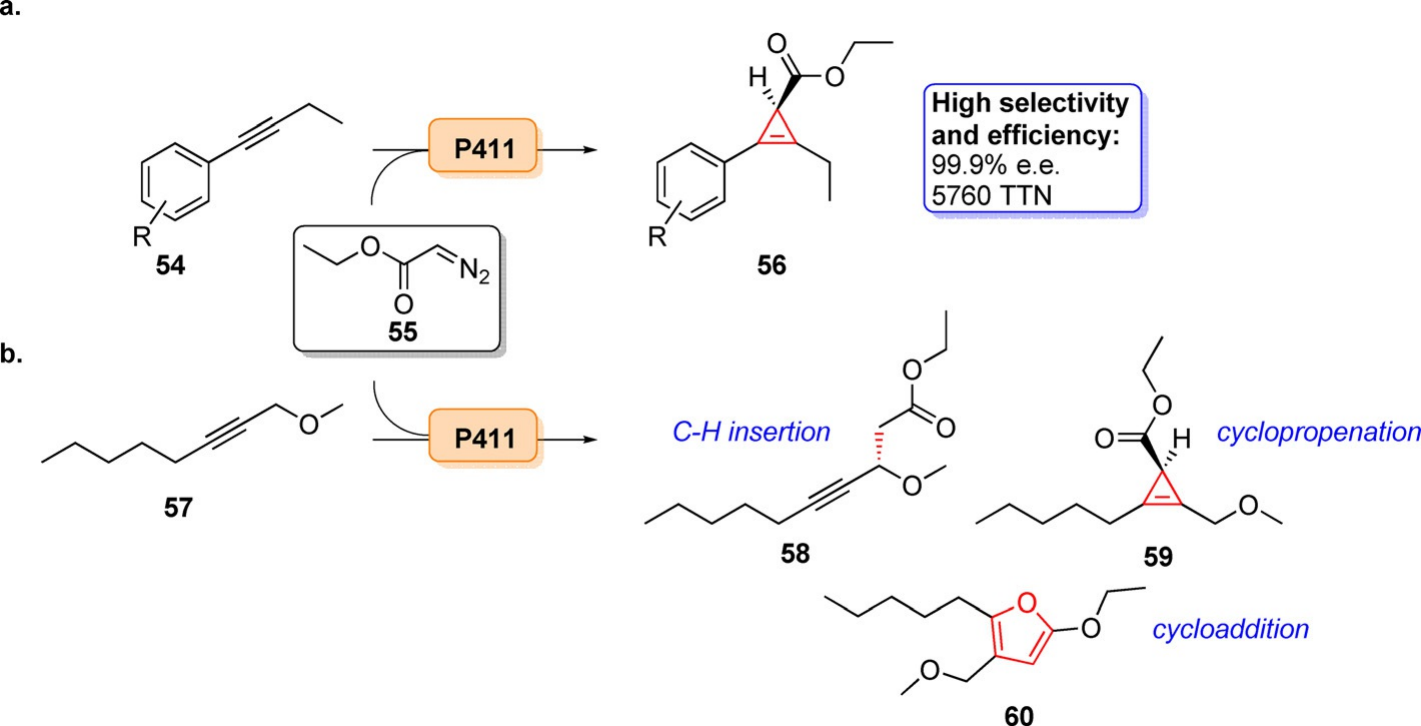
a) Enzymatic carbene transfer catalysed by a P411 variant for the cyclopropenation of internal alkynes. b) Chemoselective P411 variants are capable of either propargylic C−H insertion (**58**), cyclopropenation (**59**), or [3+2] cycloaddition (**60**).

**Scheme 10:**
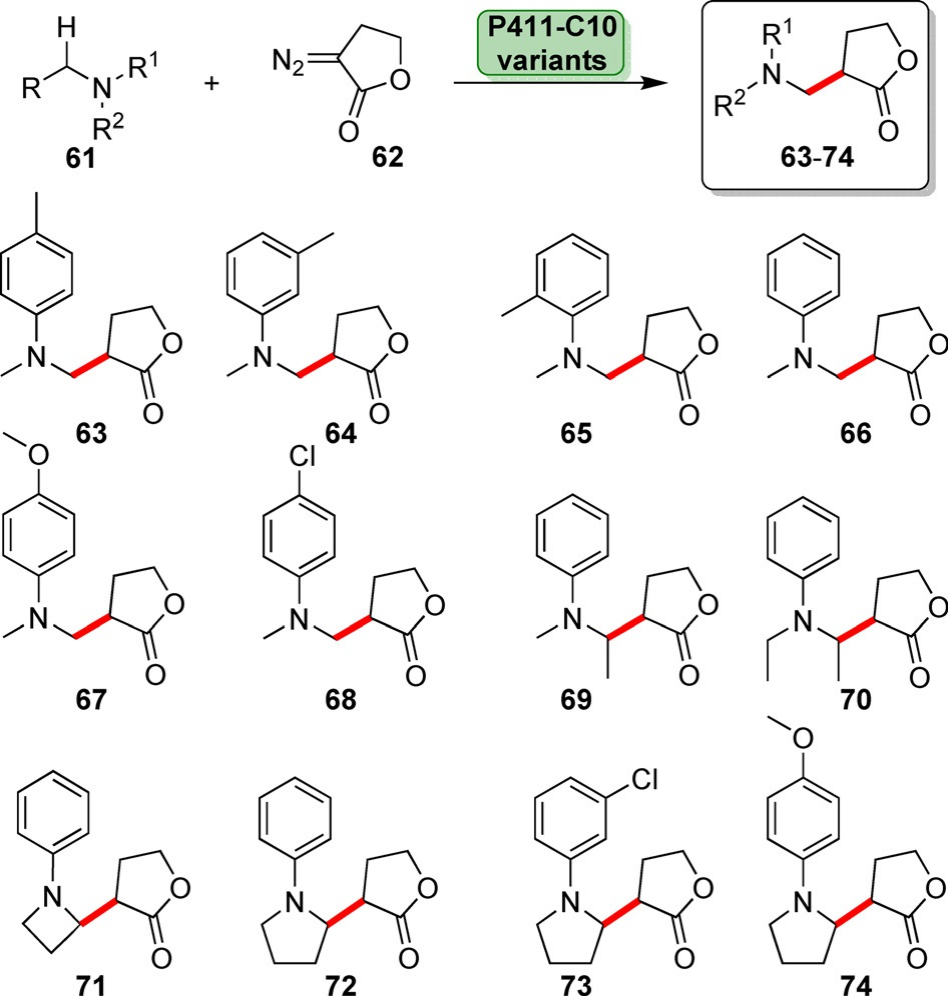
Diversity scope of a lactone–carbene insertion panel into α‐amino primary and secondary C−H bonds using engineered variants derived from the parent P411‐C10 that was previously applied in the aforementioned cyclopropenation (Scheme 9).

Recently, enantioselective cyclopropenation of internal alkynes was shown. An evolved P411 variant was able to catalyse cyclopropenation of a diverse array of alkyne substrates (**54**). A high degree of stereoselectivity (>99.9 % *ee*) of the resultant cyclopropene (**56**) was achieved by making use of the highly efficient P411 variant (TTN ≤5760, Scheme 9 a).^[83]^ P411 variants were also capable of chemoselective propargylic C−H insertion (**58**), cyclopropanation (**59**), or [3+2]‐cycloaddition (**60**, Scheme 9 b).^[83]^ Enzyme variants have recently been developed towards the stereoselective lactone carbene insertion (Scheme 10). Introducing this functionality gave rise to several analogues of sesquiterpene–lactone amine derivatives (**61**–**74**) with high enantio‐ and diastereoselectivities.^[82]^

**Figure 12:**
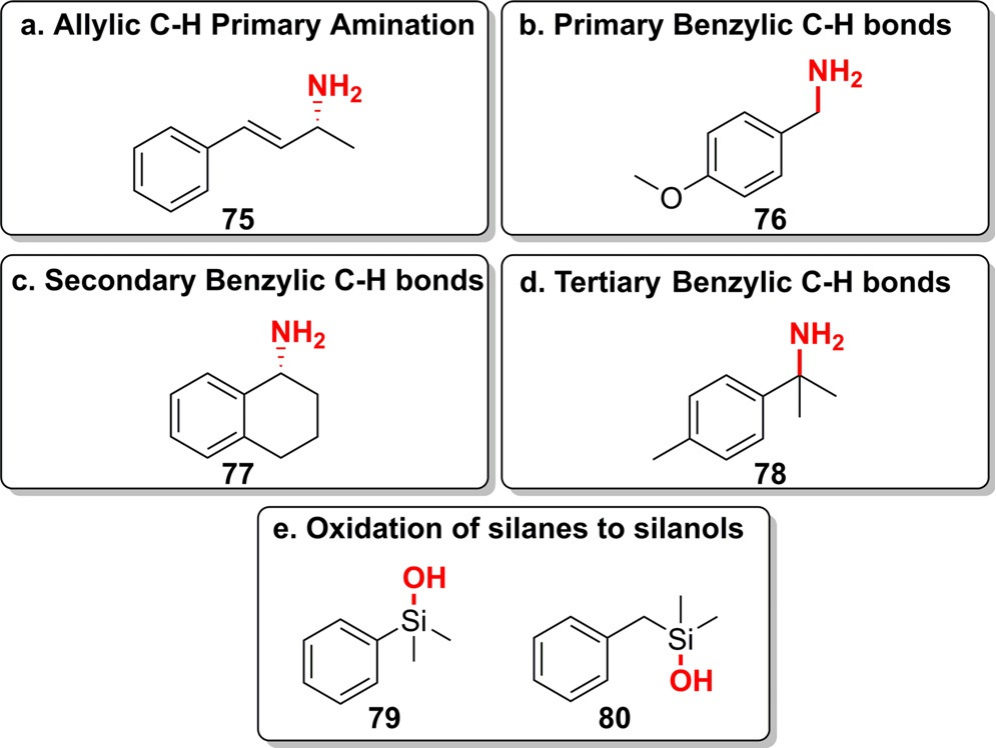
a–d) Primary amination of allylic and benzylic C−H bonds, biocatalysed via P411 variants. e) Examples of selective silane oxidation using engineered P450‐BM3 emphasising the potential to make Si‐based chemistry accessible in biocatalysis.

Furthermore, the recent engineering of P411 towards C(sp^3^)−H primary amines by highly regio‐ and chemoselective primary amination at allylic and various benzylic positions has been reported (**75**–**78**, Figure 12 a–d).^[87]^ Thus, P411 variants have opened up a variety of new‐to‐nature reactions in LSF. Complementary activities, selectivities, and efficiencies emphasise their potential towards novel approaches of functionalisation. Interestingly, the promiscuity of P450‐BM3 towards non‐native activity has recently been demonstrated by engineering the hydroxylation of silanes to silanols (**79**–**80**, Figure 12 e).^[89]^ Although there are no FDA‐approved silicon‐containing drugs,^[94]^ incorporation of silicon within drug scaffolds as a bioisostere of carbon is of increasing interest.[^95^, ^96^]

In spite of tremendous progress and a widely expanded reaction set, novel reactions in LSF have not yet found their way into drug discovery as robustness and a wide application scope still must be proven.

## 3 Biohalogenation: Diverse Strategies of Selective Carbon−Halogen Bond Formation

### 3.1 How do Halogenases Utilise Halide Salts?

Halogenation is one of the most prevalent organic reactions widely applied in syntheses of bulk and fine chemicals. Halogen atoms often have a beneficial effect on the potency and pharmacokinetic properties of drug molecules as well as being useful handles for numerous diversifications such as cross‐couplings.[^97^, ^98^] Indeed around 30 % of drugs launched during the period from 1914–2014 harboured a halogen atom.^[97]^

**Scheme 11:**
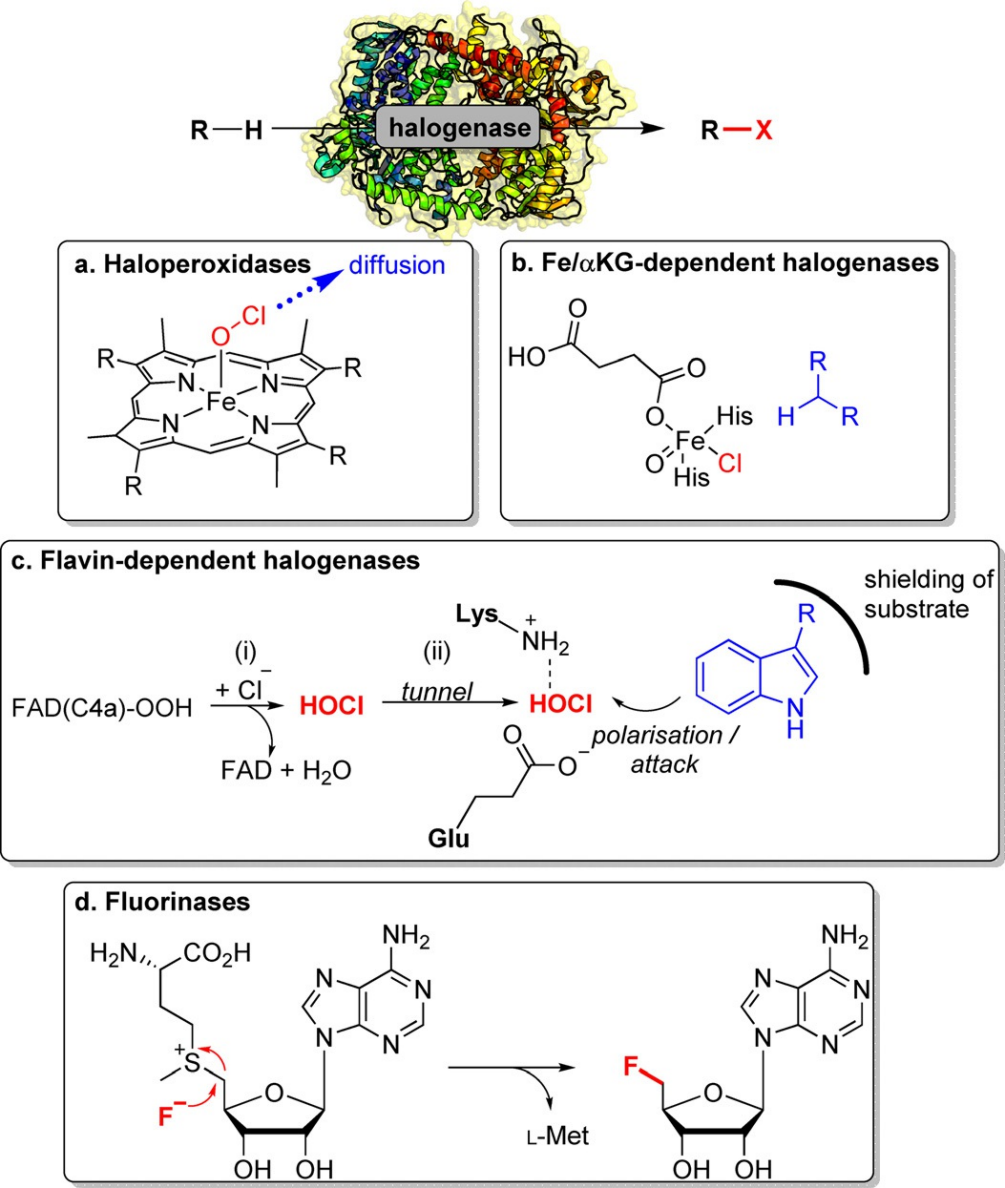
Reactive species facilitating enzymatic halogenation shown for different halogenase classes. a) Haloperoxidases: HOCl is released from the active site. b) Fe/αKG‐dependent halogenases: Non‐haem‐ferryl‐oxo species triggers radical abstraction from the substrate. c) Fl‐Hals: Hypohalous acid diffuses through a tunnel to encounter the substrate. d) Fluorinases: In a S_N_2 reaction, fluoride is transferred onto *S*‐adenosyl‐l‐methionine.

Halogenases simply utilise halide ions and molecular oxygen or hydrogen peroxide as substrates. They are subdivided into four different groups: (i) haloperoxidases (haem or vanadium‐containing enzymes); (ii) flavin‐dependent halogenases (Fl‐Hals); (iii) non‐haem iron/α‐ketoglutarate‐dependent halogenases (Fe/αKG‐Hals);[^99^, ^100^] and (iv) nucleophilic halogenases (fluorinases).[^101^, ^102^] Oxidative halogenases either form a formal “X^+^” species (X=Cl, Br, I) or a halogen radical whilst fluorinases transfer a nucleophilic fluoride ion (Scheme 11). To obtain an overview of the advancements in halogenating enzymes, Minges and Sewald published a recent update on state‐of‐the‐art applications of halogenases.^[103]^ In addition, more details on biocatalytic halogenation are summarised in Supporting Section 4 (see Supporting Information).

Since haloperoxidases do not adopt a significant role in LSF due to their inherent lack of selectivity, further discussion is omitted herein.^[104]^

### 3.2 Flavin‐Dependent Halogenases

**Scheme 12:**
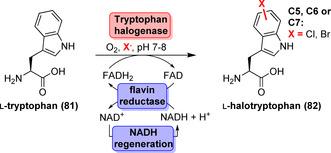
Regioselective enzymatic halogenation of l‐tryptophan (**81**) with concomitant cofactor regeneration.

Excellent regioselectivity and ambient reaction conditions are outstanding characteristics of Fl‐Hals.[^105^, ^106^] However, not all Fl‐Hals are synthetically useful since certain members require a carrier‐tethered substrate rather than a free‐standing compound.[^107^, ^108^] Today, tryptophan halogenases acting on free tryprophan as the substrate are the best studied members. The modular toolkit of regiocomplementary tryptophan halogenases has been steadily expanded over the years to selectively address the C5, C6, or C7 position of the indole moiety of l‐tryptophan (**81**) to give halotryptophan (**82**) in the presence of O_2_, a halide salt and FADH_2_ (Scheme 12).[^109^, ^110^, ^111^, ^112^, ^113^, ^114^] FADH_2_ must be provided in situ through an auxiliary reaction, e.g., by a flavin reductase, due to the inherent oxidation sensitivity of FADH_2_. Light‐driven reduction and nicotinamide mimics have also proven useful alternatives to ensure FADH_2_ supply.[^115^, ^116^] A bifunctional fusion protein consisting of halogenase and flavin reductase has been created that could foster rapid FADH_2_ exchange between the catalytic entities during catalysis.^[117]^ However, the resultant constructs exhibited impaired product yields when compared to reactions containing the two separate enzymes.

**Figure 13:**
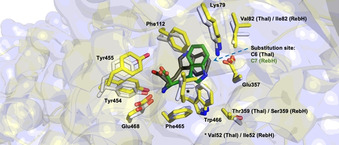
Active site overlay of RebH (PDB ID: 2OA1) and Thal (PDB ID: 6H44) highlights the modular regioselectivity of halogenases towards l‐tryptophan (**81**). Optimal tuning of both active sites results in a coplanar orientation of substrate indole side chains to each other so that either the C7 (RebH) or C6 (Thal) position is oriented towards the catalytically relevant Lys and Glu residues. Residue numbers differing between RebH and Thal are shown where necessary. Tryptophan in complex with Thal (dark grey) and RebH (green) is shown as stick models; active‐site side chains of Thal (C atoms: light grey) and RebH (C atoms: yellow) are highlighted (O atoms: red, N atoms: blue).

Crystal structures of different complementary enzymes suggest tight binding of the substrate in the active site as a well‐suited example of catalyst control, so that the C−H positions of the indole moiety are shielded by bulky residues of the protein scaffold, allowing only one carbon to be addressed by electrophilic substitution (Figure 13).[^105^, ^118^, ^119^]

Tryptophan halogenases accept a range of electron‐rich arenes and substituted tryptophans.[^120^, ^121^] Furthermore, Lewis’ group undertook extensive investigations on the substrate scope of various halogenases revealing a considerably wider substrate scope than originally assumed.^[122]^

Frese and Sewald brought the preparative application of Fl‐Hals forward by co‐immobilising the halogenase RebH with necessary auxiliary enzymes as crosslinked enzyme aggregates.^[123]^ To facilitate enzyme engineering, a fluorescence screening based on Suzuki–Miyaura cross‐coupling as reporter reaction allowed the detection of bromotryptophan via biaryl formation which serves as a directed evolution readout.^[124]^ Minges et al. carried out a sophisticated evolution campaign combining random and rational engineering to study factors influencing both thermostability and activity.^[125]^ However, despite much effort biohalogenation is still restricted by low efficiency that must be overcome to make these enzymes more valuable tools in LSF.

**Scheme 13:**
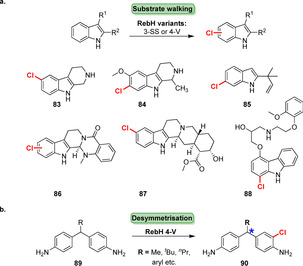
a) Late‐stage halogenation of different indole‐derived, sterically demanding compounds (**83**–**88**) achieved by stepwise directed evolution of RebH. b) Enantioselective desymmetrisation of methylenedianilines (**89**) using RebH 4‐V is provoked by the nature of substituent “R” (blue asterisk: stereocentre).

Late‐stage halogenation of large biologically active heterocycles has been achieved by a substrate walking approach. After several rounds of directed evolution and stepwise substrate modifications, bulky biologically active precursors such as alkaloids or the β‐blocker carvedilol became accessible to selective biohalogenation forming the corresponding chlorinated compounds (**83**–**88**, Scheme 13).^[126]^ Likewise, one of the mutants (RebH 4‐V) also proved useful for the desymmetrisation of methylenedianilines (**89**) by selective induction of a stereocentre remote from the halogenation site, which is hard to achieve chemically.^[127]^

**Scheme 14:**
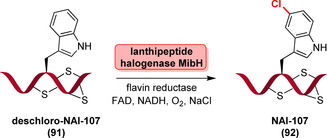
Schematic representation of late‐stage halogenation of a tryptophan residue within a lanthipeptide precursor catalysed by the non‐carrier dependent tryptophan 5‐halogenase MibH. FADH_2_ is provided by a flavin reductase by concomitantly oxidising NADH.

Recently, Ortega et al. identified an interesting tryptophan halogenase involved in the biosynthesis of a 23‐mer lanthipeptide: MibH catalyses late‐stage halogenation of a tryptophan residue within a freestanding late biosynthetic peptide precursor (**91**, Scheme 14) affording NAI‐107 (**92**).^[128]^ MibH is highly substrate specific so that even slight variations in the peptide substrate were not accepted, thus restricting its use in late‐stage halogenation of peptides.

**Scheme 15:**
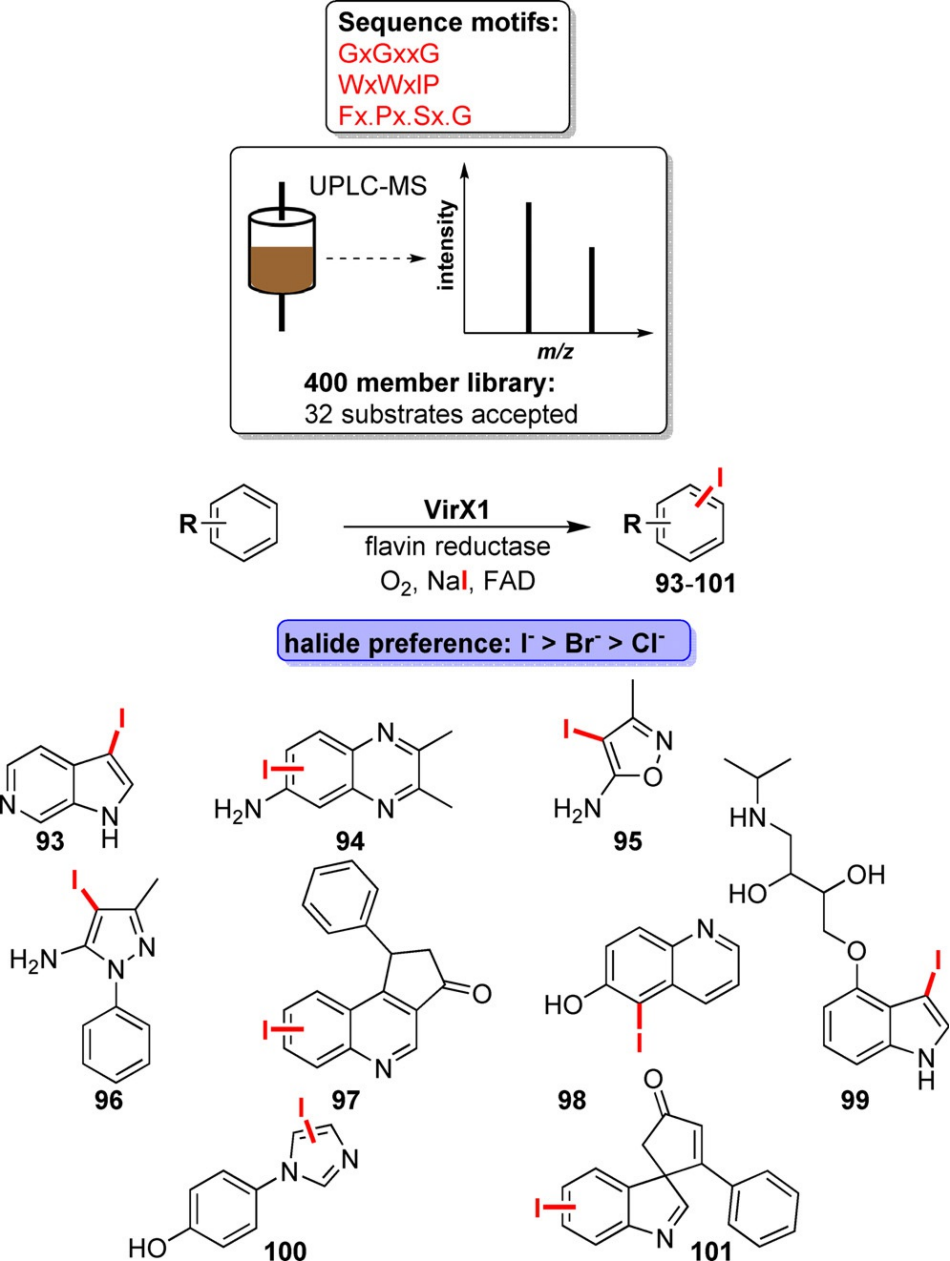
Bioinformatic halogenase screening and identification of an iodinase as described by Gkotsi et al. A sequential workflow starting from genome mining gave rise to an unprecedented iodinase; exemplary products are shown.

While the majority of LSF examples focuses on tryptophan halogenases, efforts have been undertaken to discover novel halogenases using genome mining. Accordingly, analysis of sequence similarity networks allowed the discovery of 39 new halogenases.^[129]^ This led to the discovery and closer investigation of halide‐specific halogenases such as brominases.[^130^, ^131^] Moreover, Gkotsi et al. made an outstanding discovery by finding a viral iodinase by genome mining (Scheme 15). Wide substrate profiling showed a preference for iodination of a diverse substrate panel.^[132]^ The ability to iodinate is particularly attractive for C−H activation, as aryl iodides provide more versatile starting materials in cross‐coupling reactions. Yet, it remains an unsolved question how brominases or iodinases preferably accept bulkier halides and whether the redox potential and/or steric demands are decisive factors in controlling halide preference.

### 3.3 Non‐Haem Iron/α‐Ketoglutarate‐Dependent Halogenases

**Scheme 16:**
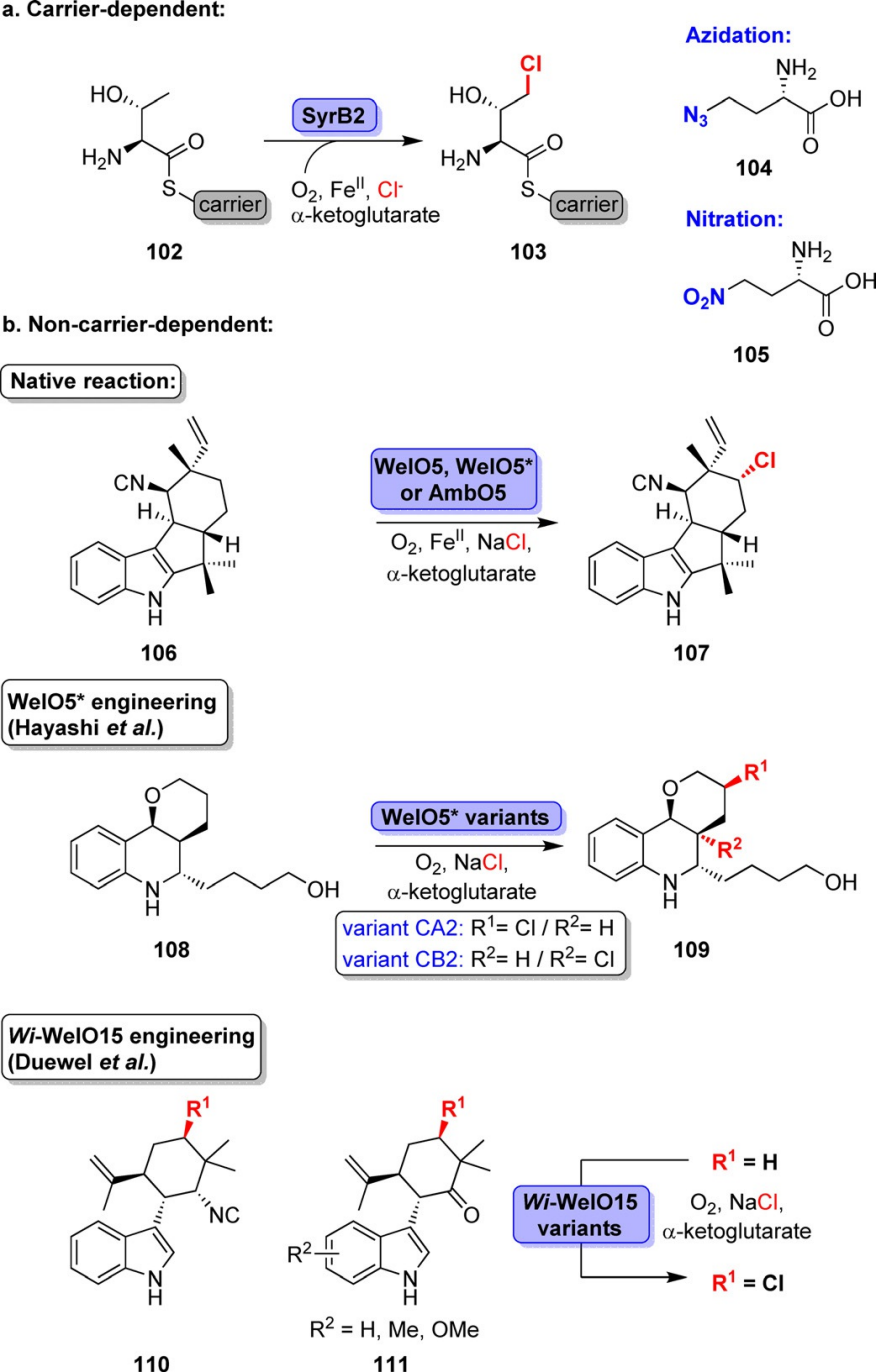
Representative examples of reactions catalysed by Fe/αKG‐dependent halogenases using carrier or non‐carrier dependent enzymes.

Fe/αKG‐dependent halogenases provide attractive tools to address less activated C(sp^3^)−H moieties, a feature that has not yet been reported for Fl‐Hals or haloperoxidases.^[133]^ The Fe/αKG‐dependent halogenase SyrB2 was the first halogenase of this type that was deeply characterised.^[99]^ Despite requiring a carrier‐tethered substrate demonstrations of in vitro activity succeeded proving halogenation of carrier‐bound l‐threonine (**102**) or l‐alloisoleucine (Scheme 16 a). Even pseudohalogens were accepted, affording the azidation and nitration of **102** to **104** and **105**.^[134]^

With the discovery of a carrier‐independent Fe/αKG halogenase, interest in this enzyme class has grown. WelO5 catalyses the selective chlorination of 12‐*epi*‐Fischer indole U (**106**) in a late stage of the biosynthesis.^[135]^ This is particularly surprising, especially with regard to the complex nature and the many similar C(sp^3^)−H positions (Scheme 16 b). Later, a few more free‐standing homologues were identified.^[135]^ AmbO5, WelO5* (or *Hw*‐WelO15), and *Wi*‐WelO15 facilitate halogenation of structurally slightly different related Fischer indoles and hapalindoles.[^136^, ^137^]

The narrow substrate scope of these enzymes is a notable drawback that has been tackled by protein engineering. Hayashi et al. described the first evolution campaign of a Fe/αKG‐dependent halogenase towards non‐indole type alkaloids.^[138]^ A martinelline‐derived fragment (**108**) served as a model substrate for evolution that started from low promiscuous activity of WelO5*. Structure‐guided evolution finally gave rise to variants CA2 and CB2 differing in regioselectivity and with pronounced increases in catalytic parameters yielding the corresponding chlorinated derivatives (**109**). However, similarly to the wild‐type enzyme, variant CA2 indicated significant hydroxylation activity. Gratifyingly, for the best variant, CB2, this side activity was abolished and a pronounced improvement in halogenation efficiency affording **109** was noted. Likewise, engineering of *Wi*‐WelO15 was reported by Hoebenreich et al. The evolved enzyme variants, created after four generations of evolution, catalysed late‐stage chlorination of non‐natural hapalindole derivatives (**110**, **111**) on a milligram scale.^[139]^

**Scheme 17:**
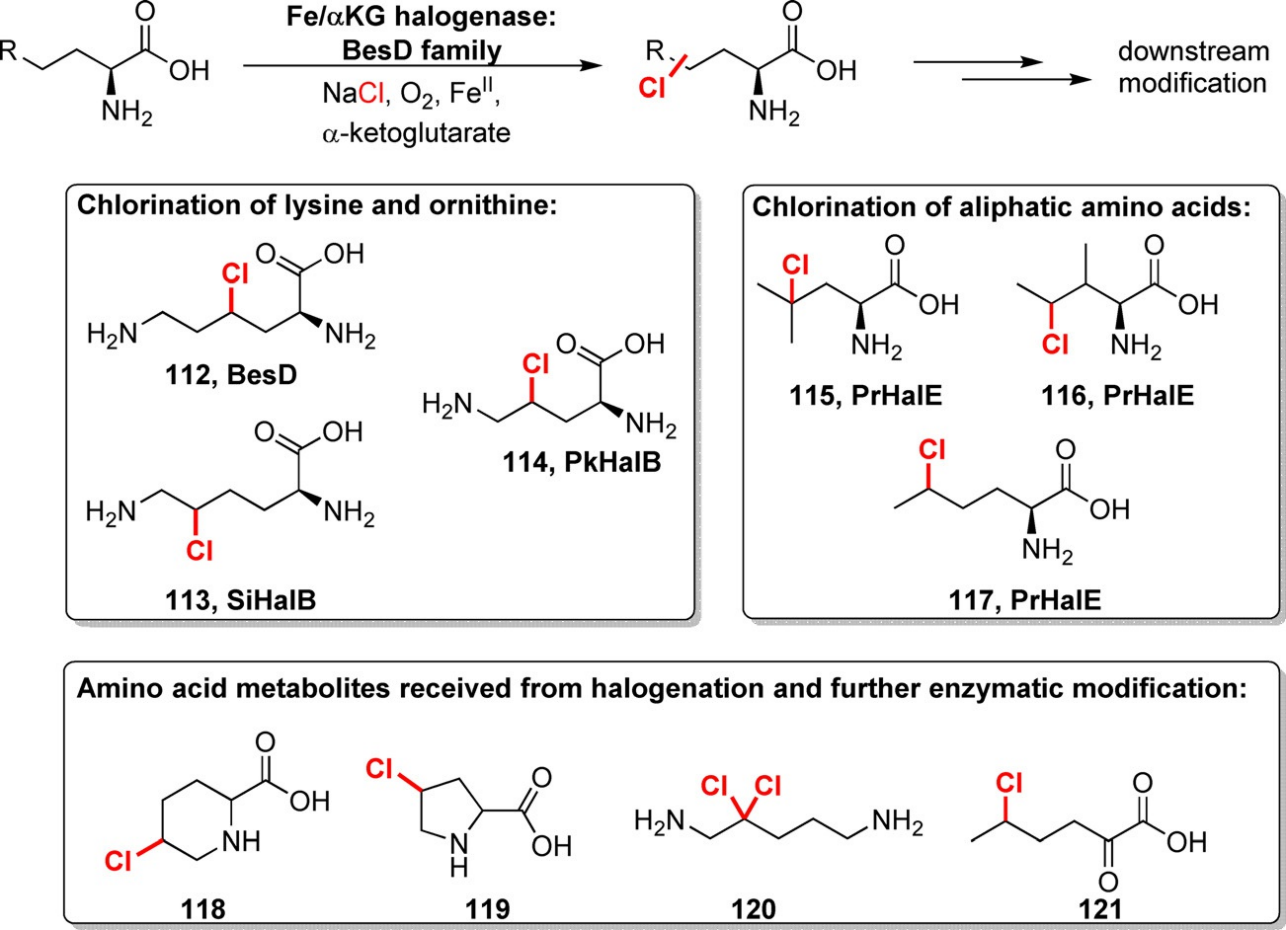
Halogenation of freestanding amino acids using halogenases of BesD family. Representative biotransformation products and the enzymes involved are depicted.

The recent discovery of Fe/αKG‐dependent amino acid halogenases further extends the synthetic utility of this enzyme class. The BesD family showed activity towards aliphatic C−H moieties of various amino acids (Scheme 17).^[140]^ Remarkably, regioselective halogenation of lysine and ornithine affording **112**–**114**, as well as of various aliphatic amino acids, e.g., leucine, isoleucine, and norleucine, succeeded. Moreover, further modification using downstream enzymes expanded the repertoire towards amino acid derivatives **118**–**121**. Biohalogenation of freestanding aliphatic amino acids has been unprecedented before and is difficult to achieve using other methods. Just recently, the first report on a nucleotide halogenase was published: The Fe/αKG‐dependent enzyme AdeV catalyses 2′‐chlorination of a deoxyadenosine moiety alongside a couple of other nucleotide derivatives albeit with lower efficiencies in the latter case.^[141]^ Undoubtedly, Fe/αKG halogenases come along with drawbacks, such as a narrow substrate scope and the fact that reactions reported until today have merely been performed on analytical or low milligram scale, which impairs their significance in biocatalysis. Even elaborate engineering has so far been difficult in broadening the substrate profile. It is important to conduct further improvements: In particular, the toolset must be extended to develop these attractive catalysts into meaningful tools for LSF.

### 3.4 Fluorinating Enzymes

**Scheme 18:**
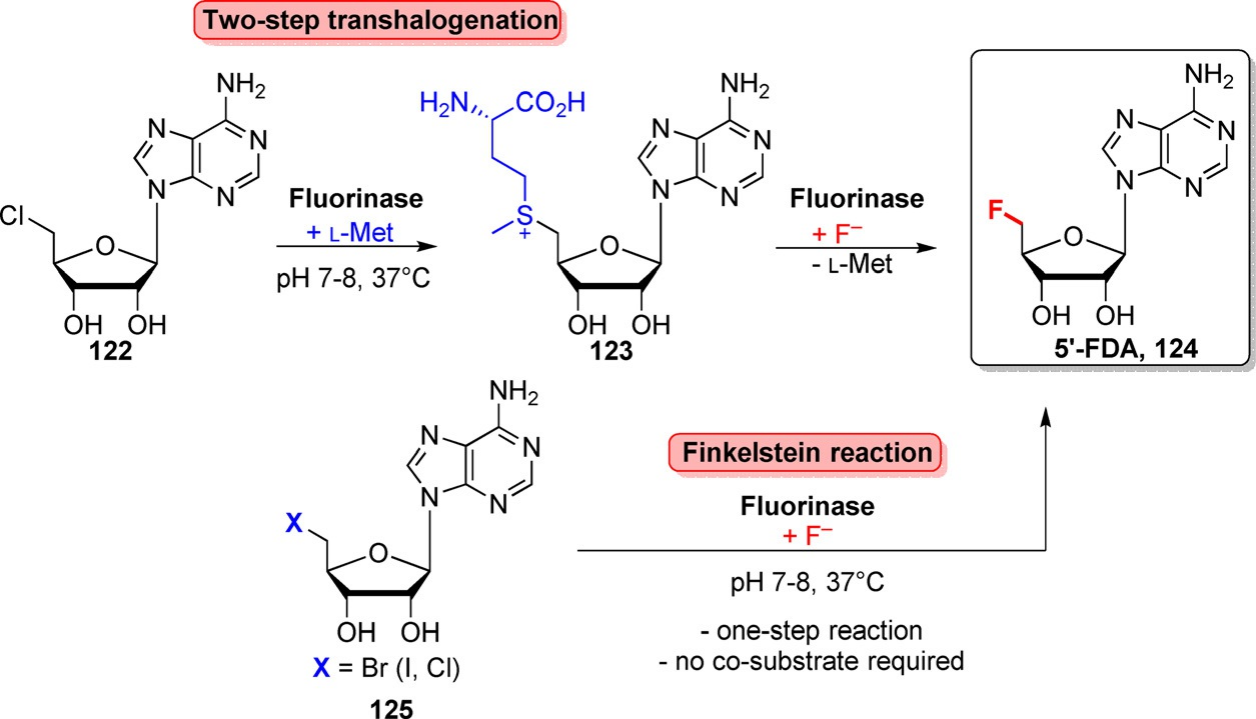
Currently applied approaches to obtain 5′‐FDA (**124**) using either a two‐step transhalogenation or a Finkelstein‐type reaction developed by O'Hagan and co‐authors.

Organofluorines represent 38 % of launched halogen containing drugs, the second most prevalent after organochlorines.^[97]^ On the contrary, fluorinated natural products are very rare and their biosyntheses generally involve an adenosyl‐fluoride synthase, usually named fluorinase.^[142]^ In 2002, the first fluorinase was discovered in the bacterium *Streptomyces cattleya*.^[143]^ Later, homologues of this enzyme followed, although the breadth is still narrow.^[142]^ Typically, the fluorinase catalyses displacement of chloride from 5′‐chloro‐5′‐deoxyadenosine (5′‐ClDA, **122**) to generate *S*‐adenosyl‐l‐methionine (AdoMet, **123**). A subsequent S_N_2 reaction gives the fluorinated metabolite 5′‐fluoro‐5′‐deoxyadenosine (5′‐FDA, **124**, Scheme 18) which turns out to be cumbersome and of low efficiency. Lowe et al. circumvented this bottleneck and further broadened the chemoenzymatic viability of fluorination by introducing a Finkelstein reaction.^[144]^ In this case, **124** could be obtained from its 5′‐brominated analogue (**125**) directly rather than in a two‐step manner.

**Scheme 19:**
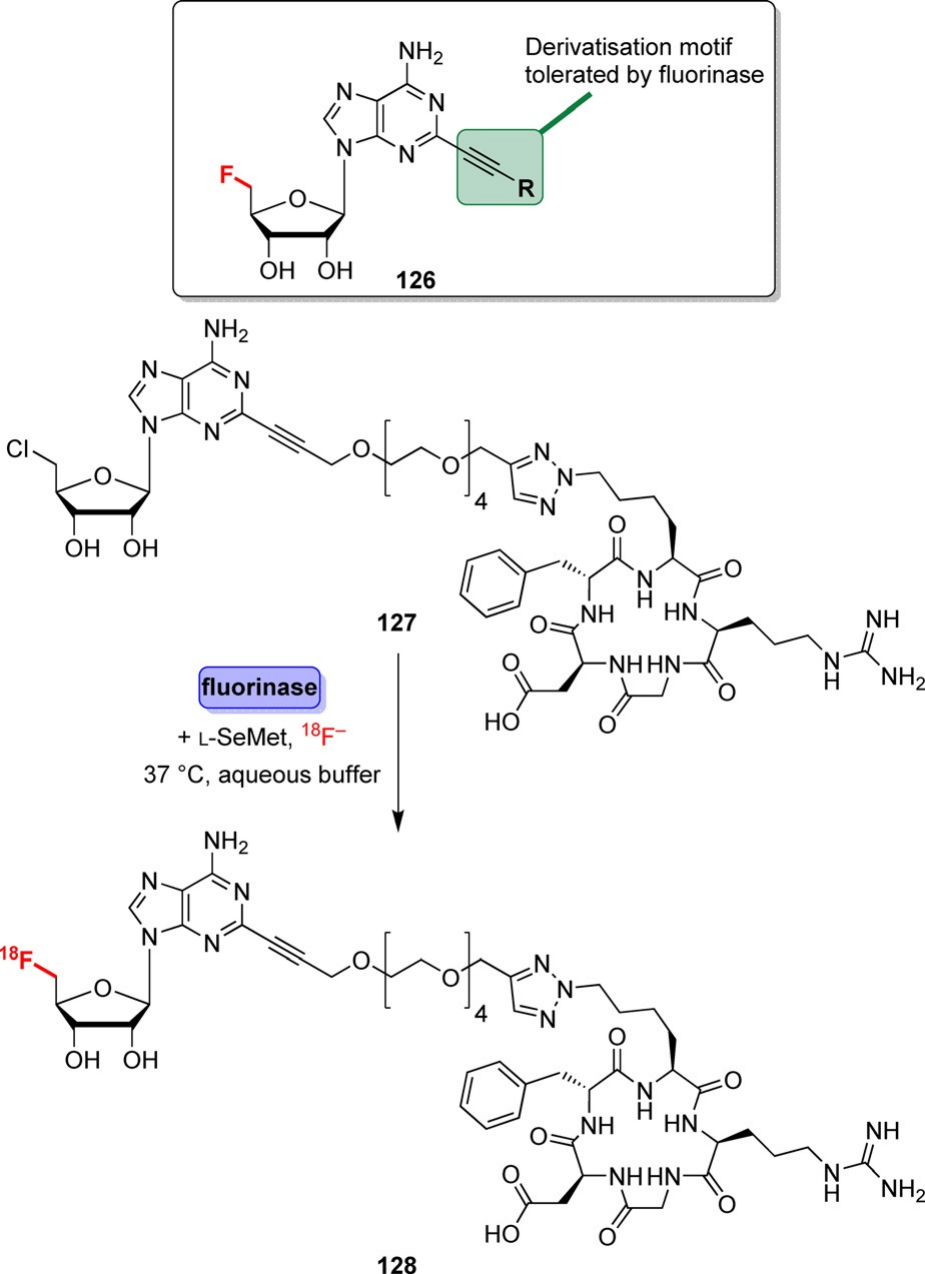
Biomolecules can be attached via the alkyne group whilst fluorination remains possible, exemplified for the late‐stage [^18^F] labelling of a cyclic RGD peptide derivative (**127**). The fluorinase catalyses the last step to introduce the radiolabel by utilising [^18^F]‐fluoride.

[^18^F]‐radiolabelling of bioactive molecules for positron emission tomography (PET) is an area where fluorinases are advantageous in achieving the site‐selective incorporation of [^18^F]‐labels.^[145]^ For instance, bulkier moieties such as the cyclic peptide c[RGDfK] can be attached via an alkyne‐derived linker (**126**) so that ^18^F‐fluorination of derivative **127** was possible (Scheme 19).^[146]^ In a similar manner, fluorinase‐catalysed transhalogenation was utilised for antibody pretargeting, as well as in labelling of a pharmacophore targeting prostate cancer tumours.[^147^, ^148^]

Despite previous approaches striving to increase fluorination efficiency by substrate optimisation and reaction engineering, the use of fluorinases in late‐stage labelling remains frustratingly rare due to low kinetic efficiencies and a very limited substrate range.

Endeavours to increase efficiency by directed evolution provided moderately improved fluorinase variants.[^149^, ^150^] In general, further efforts towards relaxing substrate specificity and simplified syntheses to obtain cofactor analogues is mandatory so that enzymatic fluorination can become a pivotal tool in the synthesis of pharmaceuticals.

### 3.5 Aryl Diversification by Combination of Biohalogenation and Cross‐Coupling Reactions

**Scheme 20:**
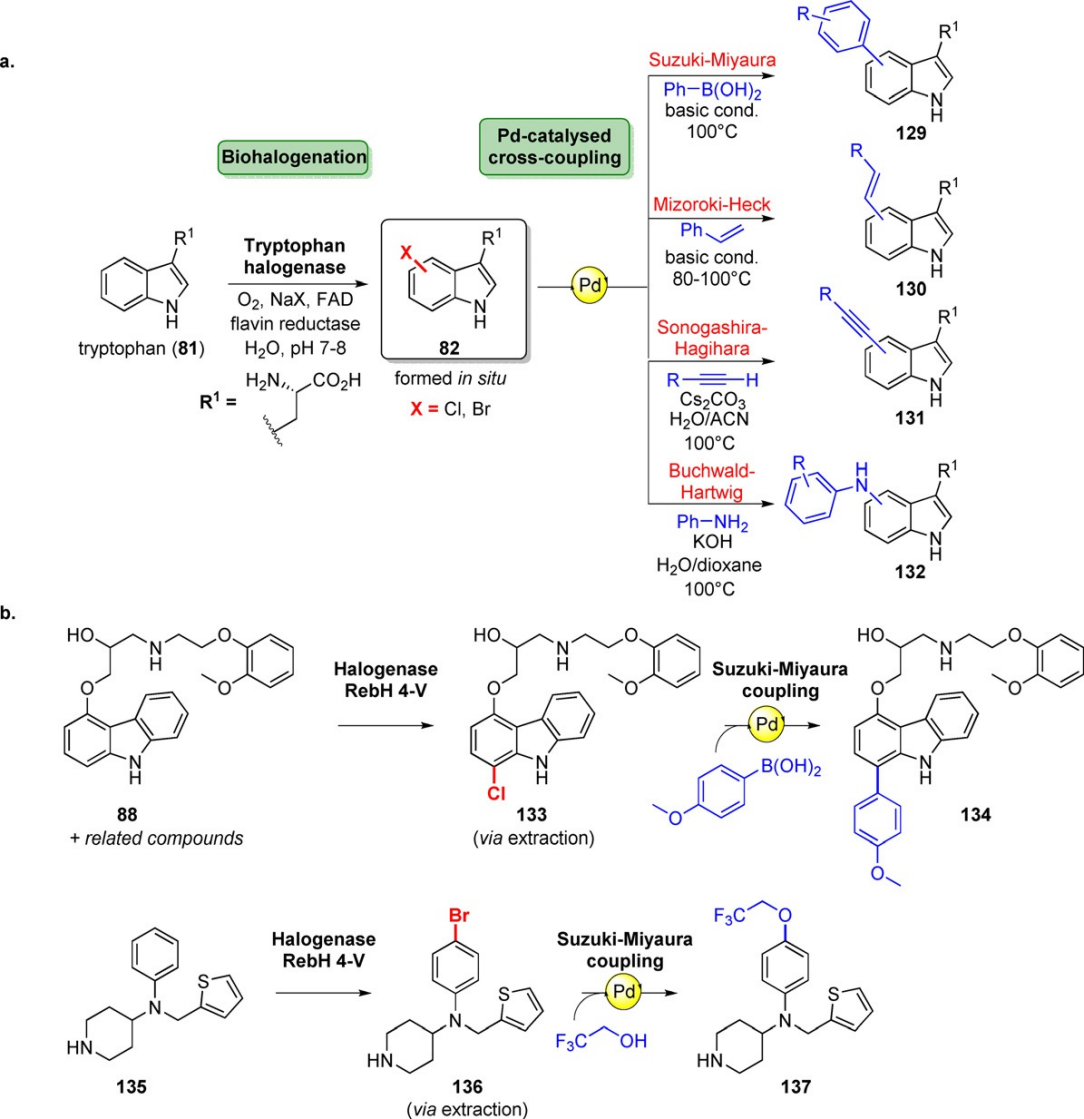
a) Types of Pd‐catalysed cross‐couplings coupled with biohalogenation for diversification of tryptophan (**81**). b) Examples of late‐stage diversification by halogenation using a RebH variant 4‐V along with Suzuki–Miyaura cross‐coupling, thus harnessing the halogenase as a handle for selective modification.

The combination of biohalogenation with Pd‐catalysed cross‐coupling is an excellent means for late‐stage C−C bond formation. Bio‐ and chemocatalytic cascade processes are particularly attractive, since the high specificity of enzymes is joined with the plethora of transformations in chemocatalysis:^[151]^ Along with a preceding enzyme‐catalysed halogenation, Suzuki–Miyaura cross‐coupling, Mizoroki‐Heck and Sonogashira reactions afforded substituted tryptophans (**129**–**132**, Scheme 20 a).[^152^, ^153^, ^154^, ^155^] By using the aforementioned RebH variant 4‐V selective halogenation of different bioactive arenes (e.g. **88**, **135**) and following cross‐couplings were carried out (Scheme 20 b), covering C−C, C−N, and C−O bond formations.^[156]^ Dachwitz et al. recently showed that Pd nanoparticles have potential as a valuable catalyst for cross‐couplings. A Suzuki reaction on both bromotryptophans and bromopeptides could be performed in water and under air at mild temperatures, an important prerequisite in chemoenzymatic cascades to overcome compatibility issues.^[157]^

**Scheme 21:**
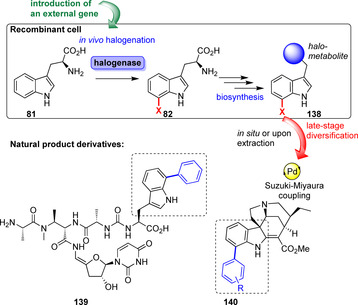
A chemogenetic approach combines halogenation of tryptophan (**81**) with natural product biosynthesis. The resultant natural product is modified by Suzuki–Miyaura cross‐coupling in the final step, as biohalogenation provides a handle for diversification of complex natural products.

Different groups succeeded in combining enzymatic halogenation and cross‐coupling in a chemogenetic approach. This allowed for the synthesis of aryl‐substituted natural products by heterologous incorporation of halogenase genes into biosynthetic pathways to obtain non‐native metabolites (**139**–**140**, Scheme 21).[^158^, ^159^, ^160^]

In vivo approaches circumvent the difficulties of handling biosynthetic enzymes to obtain complex natural product scaffolds but a sometimes complicated gene insertion and insufficient tolerance of the extrinsic gene by the host organism may eventually become problematic. Isolation of a modified natural product from the fermentation broth is often challenging as the purification results in low isolated yields. Thus, this approach currently lacks wide applicability, e.g., to obtain natural product‐inspired drug libraries.

## 4 Late‐Stage Alkylation and Acylation

C−C bond formation is fundamental in synthesis for the construction of carbon skeletons towards complex molecules. Manifold methodologies are available in chemistry, ranging from the aldol reaction to the wide field of organometallics whereas those transformations are less evolved in enzyme catalysis.^[161]^ Nevertheless, late‐stage reactions capable of carbon transfer onto multi‐functionalised scaffolds are pivotal in drug development.

### 4.1 Biocatalytic Transfer of Methyl Groups and Analogues

Notable enhancements in receptor‐binding affinity can be observed by methylation of a drug lead, a phenomenon that medicinal chemists refer to as the “magic methyl” effect.^[162]^ Selective installation of methyl groups in complex molecules can be very challenging requiring multiple chemical synthetic steps and harsh methylation reagents.^[163]^

In Nature, *S*‐adenosylmethionine (SAM or AdoMet)‐dependent methyltransferases (MTases) catalyse selective methylation of biopolymers, e.g., nucleic acids, proteins, or secondary metabolites, which exemplifies an inherent ability to perform targeted methylation. AdoMet‐dependent MTases are capable of transferring a methyl group from AdoMet (**123**) to a variety of nucleophiles (e.g. C, O, N, S, P).^[164]^ Initially, the crystal structure of catechol‐*O‐*methyltransferase (COMT) set the basis for the design of variants producing either *meta*‐ or *para*‐methylated catechols and providing building blocks towards drugs such as aliskiren and mesopram.^[165]^

**Scheme 22:**
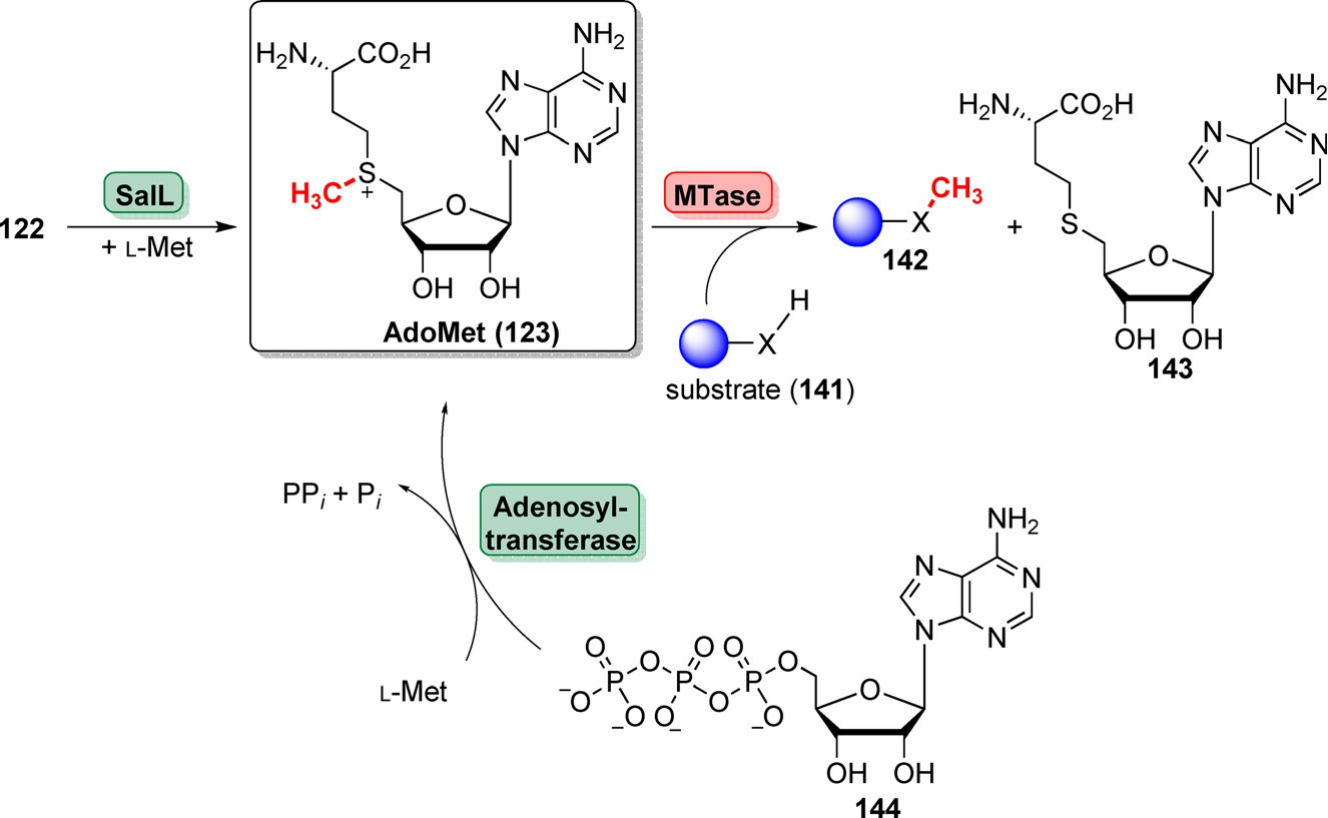
Enzymatic approaches towards generation of methyl donor AdoMet (**123**) essential for methylation using MTases. SalL catalyses the substitution of the 5′‐chloro substituent of **122** against l‐Met affording **126**. Alternatively, MATs utilise ATP (**144**) and l‐Met to yield **123**.

However, there is an ongoing demand to improve the viability of enzymatic methylation strategies. A severe bottleneck is AdoMet (**123**) due to its inherent instability (*t*_1/2_=942 min at pH 8.0 and 37 °C), cumbersome synthesis, and high cost.^[166]^ Attractive enzyme cascades for AdoMet supply were reported in the past: The enzyme SalL, naturally acting as a fluorinase (cf. Section 3.5), enables transfer of l‐Met onto 5′‐ClDA (**122**) affording **123** (Scheme 22).^[167]^ Combined with an MTase, an in situ supply of donor **123** permits methylation of a substrate nucleophile (**141**). Alternatively, methionine adenosyltransferases (MAT) can be utilised to generate **123** from ATP (**144**) and l‐Met offering the advantage of coupling AdoMet generation with an ATP recycling system and circumventing efforts to synthesise **122**.^[168]^

**Scheme 23:**
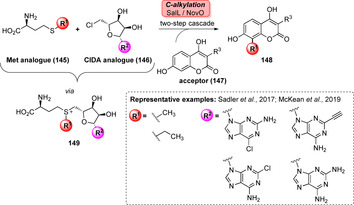
Synthesis of an array of AdoMet analogues using SalL along with Met (**145**) and ClDA (**146**) analogues. Modification of the nucleobase (R^2^) attached to the ribose moiety has been proven useful to rise cofactor stability. Alteration of the thioether chain (R^1^) permits incorporation of alkyl residues other than methyl.

AdoMet analogues provide a notable achievement towards late‐stage derivatisation, owing to increased cofactor lifetime and the ability to introduce other carbon building blocks.^[169]^ For example, a one‐pot, two‐step reaction afforded NovO‐catalysed C‐methylation using methionine (**145**) and ClDA analogues (**146**) that are accepted by SalL. Even non‐native ethylation was feasible highlighting the flexibility of this broadly studied system (Scheme 23).[^166^, ^170^, ^171^]

**Scheme 24:**
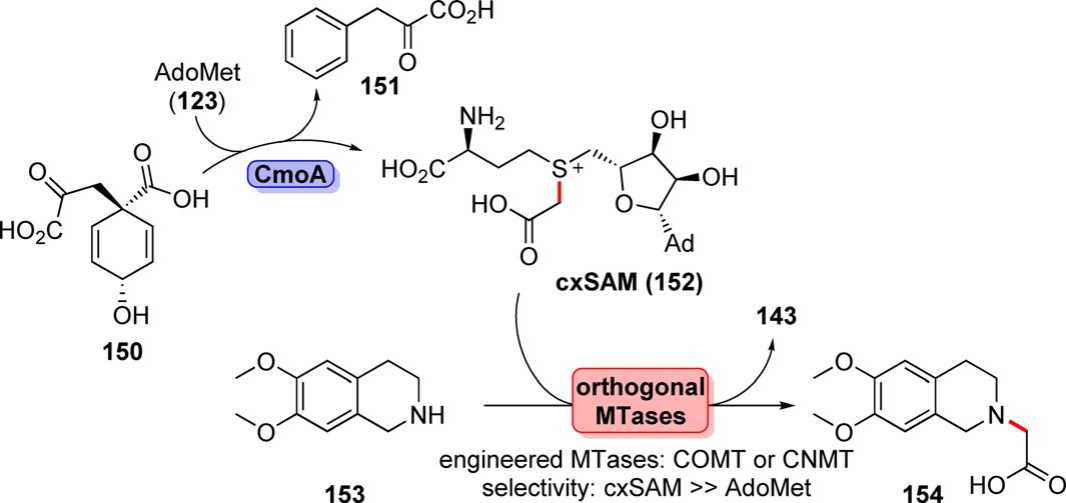
cxSAM (**152**) is generated from AdoMet (**123**) and prephenate (**150**) using the synthase CmoA. Upon engineering of orthogonal MTases, finally selective carboxymethylation was achieved.

Recently described carboxymethylation further expands the scope of carbon moieties amenable for scaffold decoration:^[172]^ The rare AdoMet derivative cxSAM (**152**) is produced by the synthase CmoA in situ from prephenate (**150**) and **123** (Scheme 24). In addition, orthogonal variants of MTases, COMT and CNMT (coclaurine‐*N*‐methyltransferase), were engineered towards higher donor specificity to suppress competing methylation whilst utilising the alkyl donor **152** to facilitate orthogonal insertion of the carboxymethyl residue into the acceptor substrate (**153**).

**Scheme 25:**
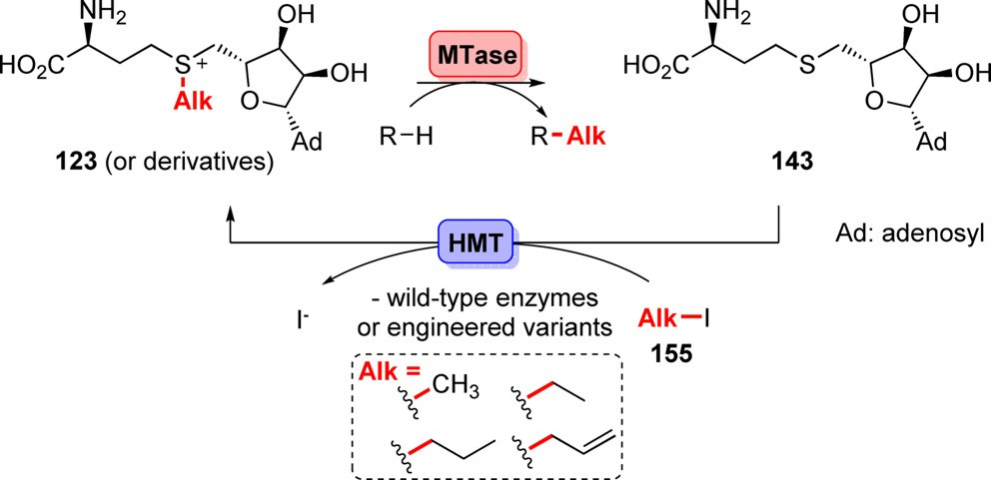
AdoMet regeneration by utilising easily available alkyl iodides (**155**) which are accepted by HMTs. An MTase enables the straightforward biocatalytic transfer of alkyl groups by making use of donor molecule **123** or its derivatives.

Liao and Seebeck recently reported on a highly innovative AdoMet recycling system that overcomes poor atom economy and the need for complex multistep reaction systems.^[173]^ A halide methyl transferase (HMT) accepts an alkyl iodide (**155**), e.g., methyl iodide (MeI), as a sacrificial methyl donor (Scheme 25): The authors showed that HMT catalyses the exergonic reaction of *S*‐adenosyl homocysteine (**143**) and MeI towards **123**. Application in a cascade using a transaminase and an MTase along with HMT afforded β‐methyl‐α‐amino acids.^[174]^ The new AdoMet generation approach was expanded in two very recent studies: Evolution of HMT succeeded so that alkyl halides other than MeI were made accessible. This notable achievement makes alternative types of enzymatic alkylations possible.^[175]^ Likewise, *N*‐methylation, ‐ethylation, and ‐propylation of pyrazoles resulted in unprecedented regioselectivities using engineered MTAses. A promiscuous HMT identified from a fungus permitted the use of various haloalkanes other than methyl iodide as the substrates, thus generating the corresponding alkylated AdoMet derivatives for transfer onto the pyrazole scaffolds.^[176]^

**Scheme 26:**
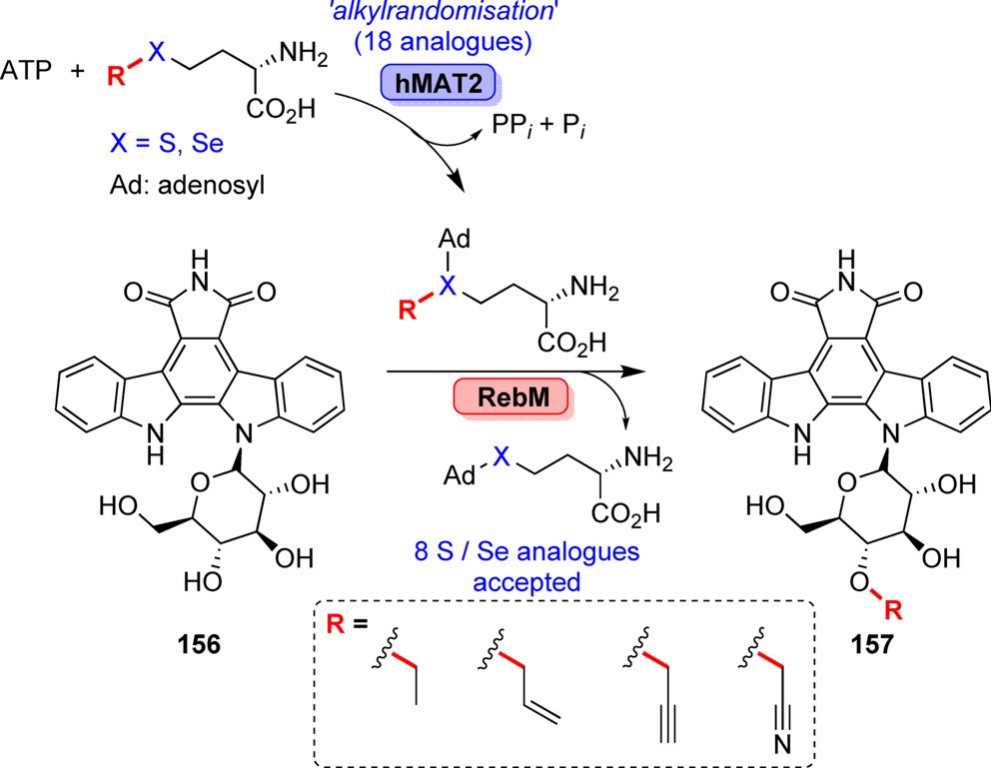
Late‐stage alkylation of indolocarbozole exemplified for a rebeccamycin derivative (**156**). Alkylrandomisation was performed by coupling the human hMAT2 with MTase RebM. Four pairs of S/Se congeners were accepted, thus forming the corresponding alkyl derivatives (**157**).

An approach for diversification called “alkylrandomisation” enabled various alkylations of a rebeccamycin derivative (**156**). 18 S or Se‐containing analogues of AdoMet were formed in a cumulative fashion. The promiscuity of human hMAT2 was exploited to obtain eight analogues of AdoMet, also including selenium derivatives that were accepted by the MTase RebM resulting in derivatives of the anti‐tumour agent (**157**, Scheme 26).^[171]^

**Scheme 27:**
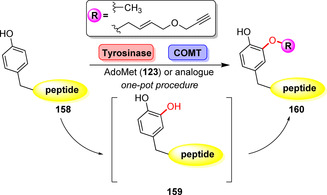
Peptide alkoxylation by combination of tyrosinase and COMT.

A combination of tyrosinase and COMT permitted late‐stage methylation of unprotected peptides:^[177]^ Directed hydroxylation of a tyrosine residue (**156**) in a peptide sequence enabled COMT‐catalysed methylation of the newly introduced hydroxy moiety in a one‐pot fashion (Scheme 27).

More recently rational engineering of fungal O‐MTases to modulate regiospecificity in the synthesis of unnatural drug‐like O‐methylated benzenediol lactone polyketides has been described.^[178]^ A notable advantage of in vivo systems is the continuous production of AdoMet (**123**). *E. coli* cells were exploited as “bio‐factories” for converting dopamine into (*S*)‐reticuline, a key intermediate in the biosynthesis of the pharmaceutically relevant benzylisoquinoline alkaloids morphine and codeine (Scheme S1, Supporting Information).^[179]^ This pathway combined five microbial or plant enzymes, three of them being different N‐ or O‐MTases. Another example is the engineering of *E. coli* cells to convert *p*‐coumaric acid into the flavonoid 7‐*O*‐methyl aromadendrin exhibiting anti‐inflammatory and anti‐cancer activity.^[180]^

### 4.2 Friedel–Crafts Alkylation and Acylation

Friedel–Crafts reactions, discovered more than a century ago, have become useful handles in synthetic chemistry due to the ability to form C−C bonds on arenes.^[181]^ Very recently, biocatalytic counterparts of these classic reactions were described, potentially offering manifold advantages such as regio‐ and stereoselectivity that often lack in traditional Friedel–Crafts syntheses. Hence, this emerging field offers a new alternative for aryl modification that has been rarely explored until now.

**Scheme 28:**
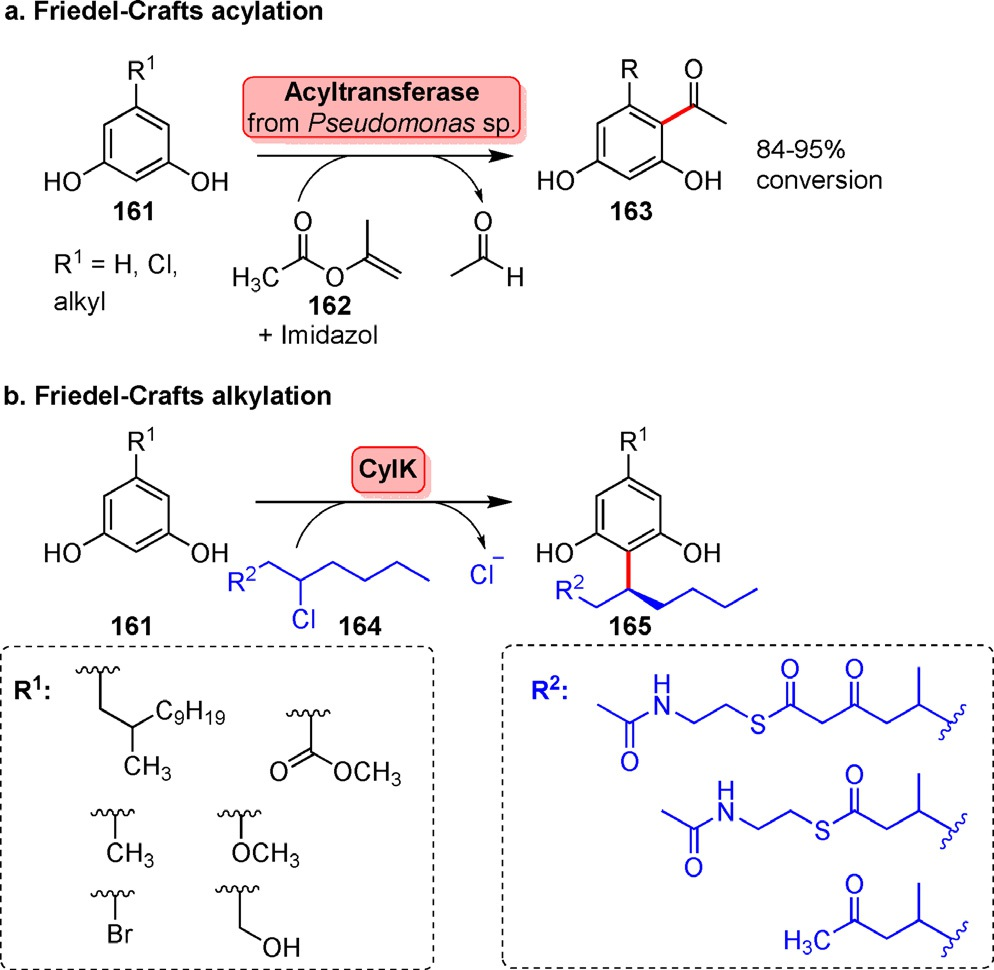
a) Friedel–Crafts acylation catalysed by an acyltransferase. Activated acyl donors were successfully transferred to resorcinols (**161**). b) Friedel–Crafts alkylation became feasible using the C−C coupling enzyme CylK.

A recent example of Friedel–Crafts acylation was reported by Kroutil's group. A bacterial multicomponent acyltransferase from *Pseudomonas protegens* (PPATase) was able to catalyse the regioselective C‐acylation of phenol derivatives, such as the electron‐rich resorcinol derivatives (**161**) by using common acyl donors (e.g. **162**, Scheme 28 a).^[182]^ Only recently, an example of an enzyme capable of direct enzymatic Friedel–Crafts alkylation was described. Natively, CylK, originating from cilindrophane biosynthesis, catalyses the formation of an aryl−alkyl linkage to afford the complex natural product.^[183]^ Its biocatalytic utility was recently exemplified showing that CylK facilitates C‐alkylation of resorcinols.

Based on this, coupling of different functionalised alkyl building blocks succeeded in C2‐position. As evidenced from these examples, further investigations are necessary to extend these novel, yet promising transformations towards substrates other than compound **161** towards late‐stage aryl functionalisation with greater utility (Scheme 28 b).^[184]^

### 4.3 Pictet–Spengler Reaction

The Pictet–Spengler reaction (PSR) is particularly useful for assembling heterocyclic scaffolds in drug design to receive differently decorated bicyclic motifs that are of interest for structure–activity relationship (SAR) studies.^[185]^ Condensation between an electron‐rich arylethylamine and an aldehyde or ketone is followed by ring closure of the intermediary iminium ion under acidic conditions. Alkaloids, tetrahydroisoquinolines (THQ) and β‐carbolines can be obtained stereoselectively, providing important natural products or pharmacological scaffolds.[^186^, ^187^]

**Scheme 29:**
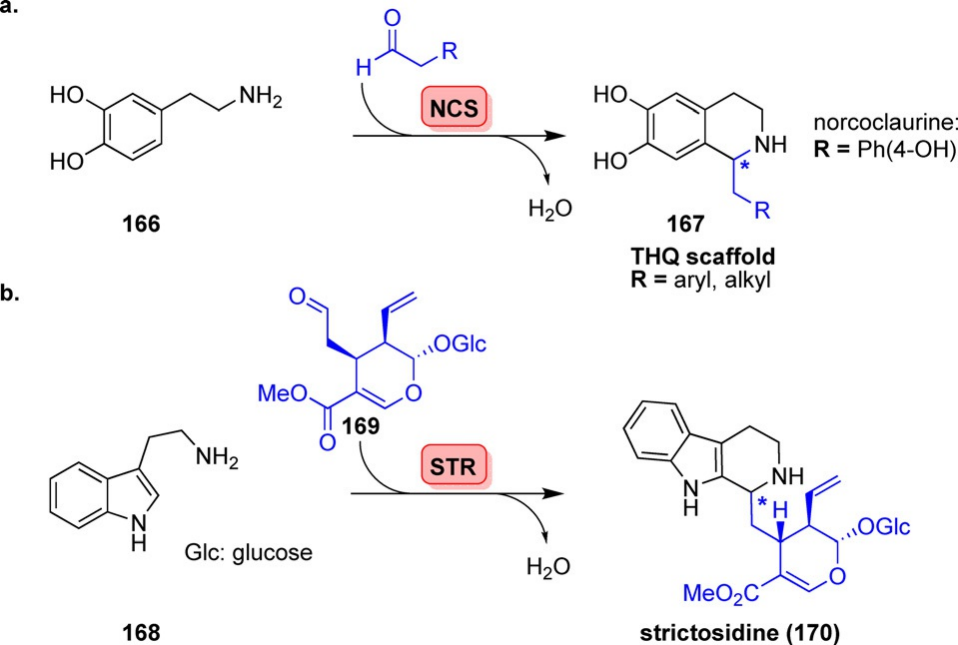
Enzymatic Pictet–Spengler reaction. a) Condensation of dopamine with aldehydes yields different THQs (**167**). b) Stricosidine (**170**) is synthesised by condensation between tryptamine (**168**) and secologanin (**169**).

Norcoclaurine synthase (NCS) and strictosidine synthase (STR) constitute well examined Pictet–Spenglerases (PSases) with regard to biotechnological applications (Scheme 29 a).^[187]^ (*S*)‐Norcoclaurine (**167**) is obtained from the condensation of dopamine (**166**) and 4‐hydroxyphenylacetaldehyde catalysed by NCS.^[188]^ The PSase STR catalyses the cyclisation of tryptamine (**168**) with secologanin (**169**) giving the 1,2,3,4‐tetrahydro‐β‐carboline (THBC) scaffold as found in the indole alkaloid (*S*)‐strictosidine (**170**, Scheme 29 b).^[189]^

**Scheme 30:**
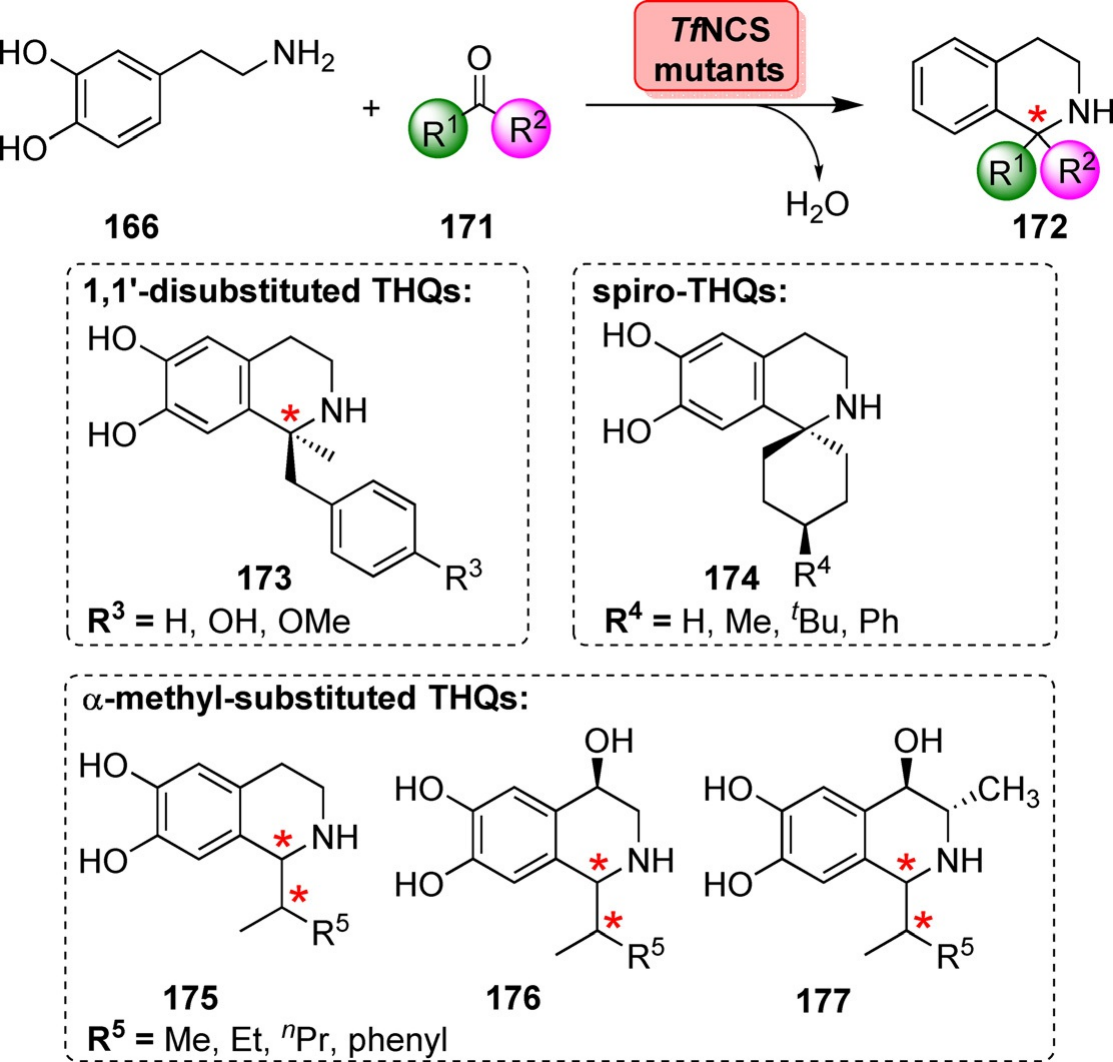
Engineering of T*f*NCS expanded the substrate scope towards disubstituted, spiro‐ and α‐methyl THQs. Representative products are shown.

To introduce diversity the substituent located at C1 of the heterocycle can be varied by choosing the desired aldehyde component.^[190]^ Broader substrate profiling further revealed that different substituents, predominantly phenyl and alkyl, could be introduced with high enantioselectivities.^[191]^ Hailes’ and Ward's groups recently envisaged developing PSases towards bulkier ketone substrates (**171**).^[192]^ A truncated NCS (Δ29*Tf*NCS) with low promiscuous activity against 4‐hydroxyphenyl acetone was rationally engineered to yield 1,1′‐disubstituted THQs (**173**) from methylketones (Scheme 30).

Interestingly, spiro‐fused derivatives (**174**) were obtained by coupling dopamine with cyclohexanones. This transformation provides a particularly interesting means for scaffold diversification. Additionally, the substrate scope could be expanded towards α‐substituted aldehydes affording THQs **175**–**177** with remarkable diastereoselectivities when building up two stereocentres.^[193]^

**Scheme 31:**
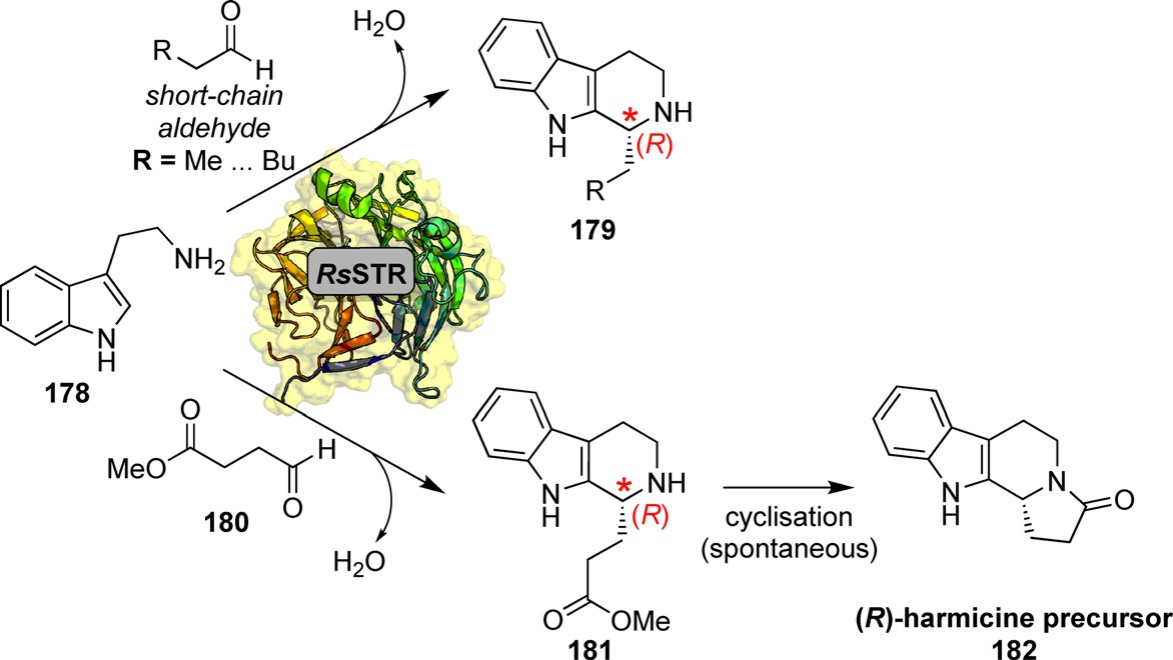
Condensation of tryptamine with short‐chain aldehydes results in unexpected (*R*)‐configuration of the products whilst (*S*)‐configuration prevails for bulkier aldehyde substrates.

Early studies on the substrate scope and active‐site engineering of STRs indicate the enzyme's promiscuity towards substituted tryptamines and different aliphatic or aromatic aldehydes albeit with lower efficiencies compared to the native aldehyde (**169**).[^194^, ^195^, ^196^] Recently, expression optimisation and screening of different STRs further extended the scope towards short‐chain aldehydes. For *Rs*STR an unexpected (*R*)‐configuration of the resultant THBCs (**179**) was found whilst (*S*)‐configuration prevailed for bulkier aldehydes (Scheme 31). Structural studies and modelling unveiled a rationale for this peculiar selectivity switch: Smaller aldehydes are bound in an inverted orientation, suggesting the different stereopreference is governed by the aldehyde.^[197]^ This selectivity feature was exploited to synthesise an (*R*)‐harmicine precursor (**182**) from **178** and aldehyde **180**.^[198]^

## 5 Selective Enzyme‐Catalysed Amide Bond Formation

### 5.1 Amide Synthesis in Nature

Amide bonds are recognised as the most prevalent motifs found in drugs as revealed by a recent analysis of functional groups found in medicinal chemistry papers.^[199]^ Not surprisingly, a survey in 2011 attributed 16 % of all reactions applied in medicinal chemistry to amide couplings.^[200]^ Numerous approaches are known and continuous development towards selective and less toxic amidation procedures is ongoing. Typical approaches of carboxylic acid activation require hazardous conditions, e.g., formation of acyl chlorides, use of carbodiimides or uronium reagents, suitable protecting groups, as well as toxic, non‐eco‐friendly solvents.^[201]^ These shortcomings are an incentive to develop new reactions that provide amides selectively under benign conditions.

**Scheme 32:**
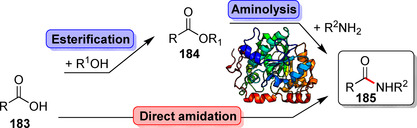
Common strategies of enzymatic amide bond formation starting from a carboxylic acid (**183**). Hydrolase‐catalysed aminolysis requires previous esterification whilst in a direct approach, the carboxylic acid serves as a substrate that is directly converted into the amide (**185**).

Apart from peptide bonds as central binding motifs in proteins, a huge number of enzymes facilitates the formation of this important connection in natural products which gain increasing attention in synthetic organic chemistry. In general, hydrolases, some transferases, and ATP‐dependent ligases are in principle capable of catalysing amidations. Predominantly, hydrolase‐based approaches proceed via aminolysis often preceded by an esterification (Scheme 32). The recently published lipase SpL that acts on free carboxylic acids and amines to directly afford amides in organic solvent/water mixtures is a rare exception (Scheme S2, Supporting Information).^[202]^ An overview on notable examples on hydrolase‐catalysed amidations using lipases and penicillin G acylases is found in Supporting Section 6 (see Supporting Information). Transferases facilitate the transfer of activated acyl donors, while ATP‐dependent transformations can directly amidate carboxylic acids under benign conditions in water.^[203]^ Herein, the main emphasis is put on direct approaches facilitating amide bond formation in the context of late‐stage modifications.

**Scheme 33:**
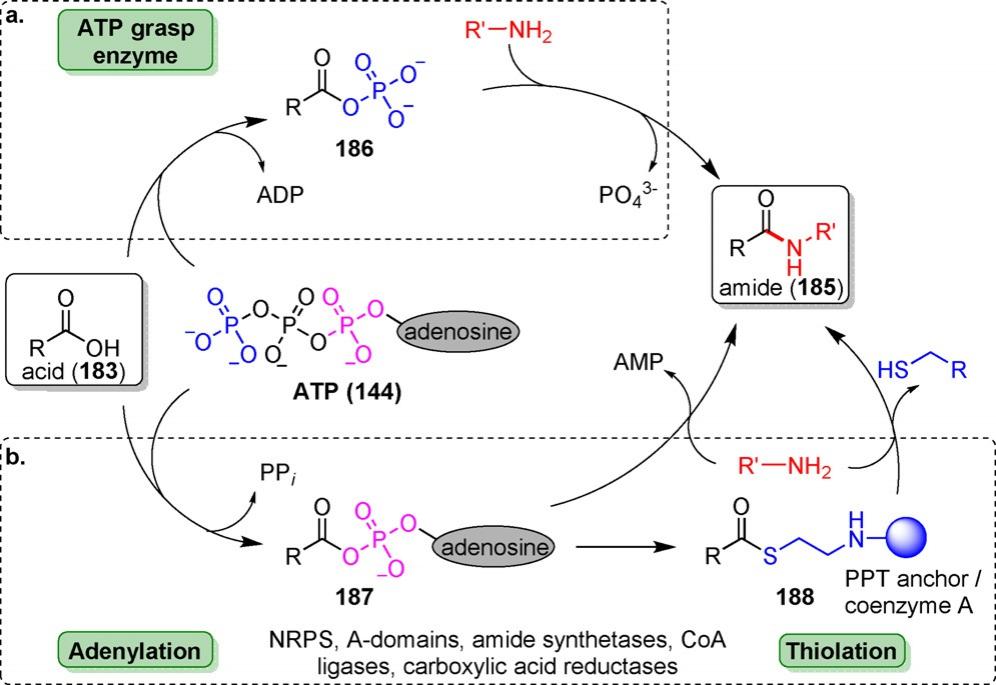
Activation of carboxylate group by ATP‐dependent enzymes. Two approaches to utilise ATP predominate: a) In ATP grasp enzymes, the γ‐phosphate is transferred upon nucleophilic attack of carboxylate leading to a mixed anhydride (**186**). b) Adenylation domains result in an acyladenylate (**187**). Depending on the enzyme the activated species is directly attacked by an amine or undergoes intermediary thioester formation. NRPS: non‐ribosomal peptide synthetase.

In recent years, manifold enzymes part of the ANL (acyl‐CoA synthetase, non‐ribosomal peptide synthetase, luciferase) superfamily of adenylating enzymes have been made available. These ATP‐dependent enzymes adopt a pivotal role and form a highly diverse enzyme class: All members have in common that ATP is utilised to facilitate activation of the carboxylic acid (**183**) prior to acyl transfer. In non‐ribosomal amide synthesis, amidation proceeds via a three‐step procedure: (i) adenylation; (ii) thiolation; and (iii) condensation (Scheme 33).^[204]^ ATP‐grasp enzymes form an acylphosphate intermediate (**186**) as the carboxylate attacks the γ‐phosphate group of ATP, while adenylation domains (A‐domains) and amide synthetases catalyse the formation of an intermediary acyladenylate (**187**). Afterwards, attack by the amine occurs directly or via a thioester intermediate (**188**).^[205]^

### 5.2 Direct Amide Bond Formation: Examples and Recent Developments in Late‐Stage Functionalisation

**Scheme 34:**
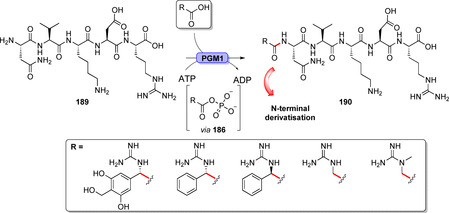
N‐terminal derivatisation of pheganomycin derivatives using the peptide ligase PGM1. The enzyme catalyses selective acylation of the peptide's N‐terminus.

l‐Amino acid ligases are predominant members of the ATP‐grasp enzyme family. They often facilitate the formation of peptide bonds in biosynthesis. A rather promiscuous ATP‐grasp enzyme was characterised from the biosynthesis of tabtoxin: TabS is capable of forming various dipeptides from unprotected amino acids. 136 different amino acid combinations led to the formation of the corresponding dipeptides including non‐proteinogenic amino acids.^[206]^ Although several other l‐amino acid ligases have been characterised, their applications remain scarce due to pronounced lower productivity compared to conventional peptide synthesis and high substrate specificity.[^207^, ^208^, ^209^, ^210^] A significant finding towards LSF was the identification of the grasp enzyme PGM1 from the biosynthetic pathway of the peptide antibiotic pheganomycin (**190**). PGM1 was shown to catalyse the selective N‐terminal coupling of the biosynthetic precursor peptide (**189**) to different substituted acetic acids, hence providing a potentially suitable means for peptide modification (Scheme 34).^[211]^

**Scheme 35:**
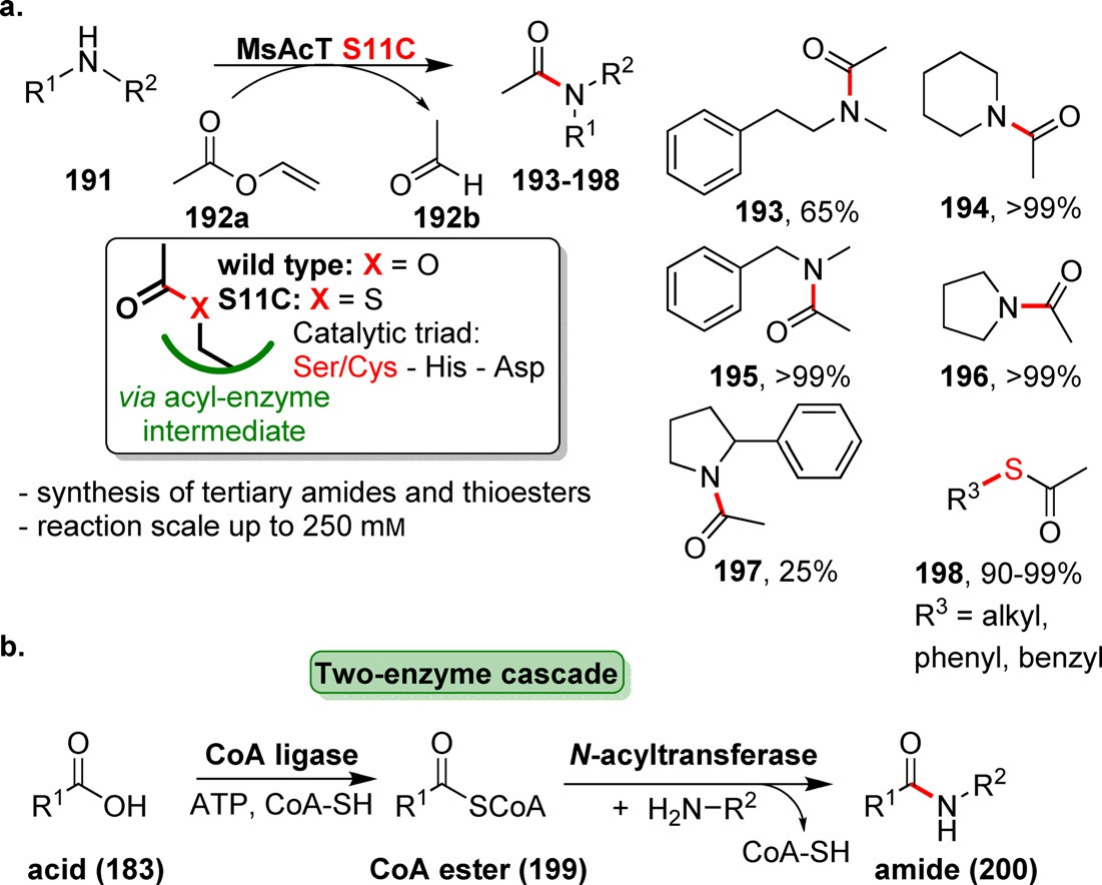
a) *N*‐Acyltransferase MsAcT S11C accomplishes difficult acetylations with high substrate concentration due to exchange of active‐site Ser against Cys forming a covalent thioester intermediate. b) Combination of CoA ligase and *N*‐acyltransferase affords a broad amide scope.

*N*‐Acyltransferases enable the transfer of acyl groups from activated esters onto various amines. For example, acylation of amines in water employing vinyl esters was demonstrated for the transferase MsAcT by Paradisi and co‐authors.^[212]^ Just recently the reactivity of MsAcT was considerably tuned by mutating the active‐site serine, part of the catalytic triad, into a cysteine residue.^[213]^ Variant S11C afforded synthesis of thioesters and challenging tertiary amides with an industrially viable substrate loading (Scheme 35 a). Furthermore, Lovelock et al. reported on a two‐enzyme platform that combines CoA ligases and acyltransferases (Scheme 35 b).^[214]^ A coenzyme A thioester (**199**) is formed from acid **183** by a ligase and attacked by the amine nucleophile using a *N*‐acyltransferase in the second step. Provided that suitable pairs of ligase and acylase can be found, a variety of amidations are covered without making use of previously activated acyl donors.

Adenylation domains (A‐domains) are widespread in NRPS systems providing an entity for ATP‐dependent carboxy group activation. Stand‐alone A‐domains from an NRPS system responsible for the biosynthesis of streptothricin antibiotics in *Streptomyces* sp. were shown to act as self‐sufficient biocatalysts catalysing adenylation of the β‐amino acid l‐β‐lysine which subsequently undergoes attack either by a PCP (peptidyl carrier protein) domain or carrier‐bound l‐β‐lysine to afford an oligopeptide.^[215]^ Also, A‐domain‐catalysed amidation was shown for fatty acids towards a range of amines, also enabling the synthesis of rare *N*‐acyl histidines alongside other acylated compounds.^[216]^ Diversification of tryptophan succeeded by using the A‐domain of tyrocidine synthetase (TycA).^[217]^ A rather specialised substrate scope often limits the widespread use of A‐domains from NRPS systems.

**Scheme 36:**
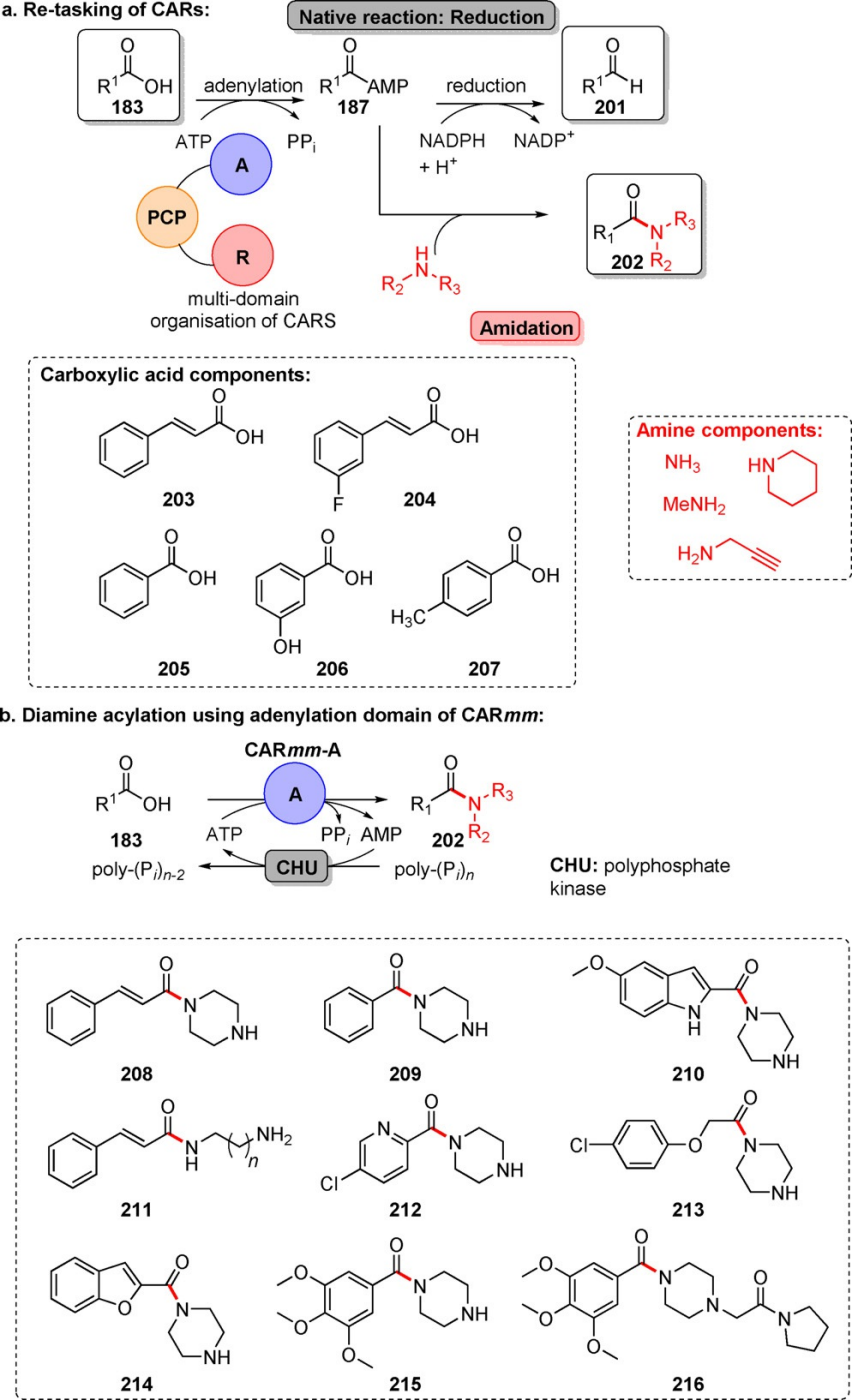
a) Carboxylic acid reductases can be used for amide bond formation rather than reduction of a carboxylic acid. b) Solely the A‐domain is sufficient to enable amidation as shown by using a truncated CAR (CAR*mm*‐A) along with a kinase for concomitant ATP regeneration.

In contrast, carboxylic acid reductases (CARs) natively catalyse the reduction of carboxylic acids (**183**) into the corresponding aldehyde (**201**).[^218^, ^219^] Structural studies and engineering revealed CARs to be multi‐domain enzymes consisting of a distinct A‐, PCP‐ (peptidyl carrier protein, containing a phosphopantetheine arm) and a reduction domain (Scheme 36 a).^[220]^ Wood et al. showed that CARs are capable of amidation when employing an excess of amine rather than the co‐substrate NADPH, so that the reduction function is abolished.^[221]^ Hence, an array of benzoic and cinnamic amides was synthesised. In a follow‐up study this concept was further streamlined by making use of a truncated CAR variant (CAR*mm*‐A) consisting of merely the A‐domain. The authors showed that CAR*mm*‐A performs selective mono‐acylation of diamines without the need for protecting groups. An array of carboxylic acids with loadings up to 10 mm was utilised in a facile one‐step amidation yielding various amides (**208**–**216**), e.g., the vasodilator cinepazide (**216**, Scheme 36 b).^[222]^

**Scheme 37:**
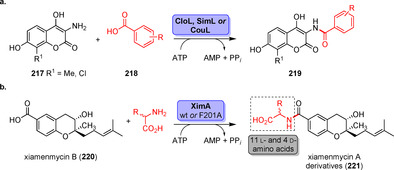
a) Amide coupling of aminocoumarins (**217**) to obtain modified acylated aminocoumarins (**219**). b) Xiamenmycin derivatisation by selective coupling of amino acids to **220** using XimA wild type or a mutant.

Despite their current narrow substrate scope and difficult handling due to weak expression and solubility, amide bond forming synthetases are receiving increased attention for late‐stage modification. Wessjohan and co‐authors reported on the homologous amide synthetases CloL, SimL, and CouL utilised in a modular fashion to modify aminocoumarin (**217**). A small library of derivatives on a milligram scale was synthesised using this system (Scheme 37 a).^[223]^ The amide synthetase XimA was shown to catalyse the last biosynthetic step by coupling l‐threonine to the free carboxylic acid precursor (xiamenmycin B, **221**) in xiamenmycin A biosynthesis (Scheme 37 b). The authors successfully expanded the amino acid scope by rational mutant design from l‐threonine towards 11 l‐ and 4 d‐amino acids.^[224]^

**Scheme 38:**
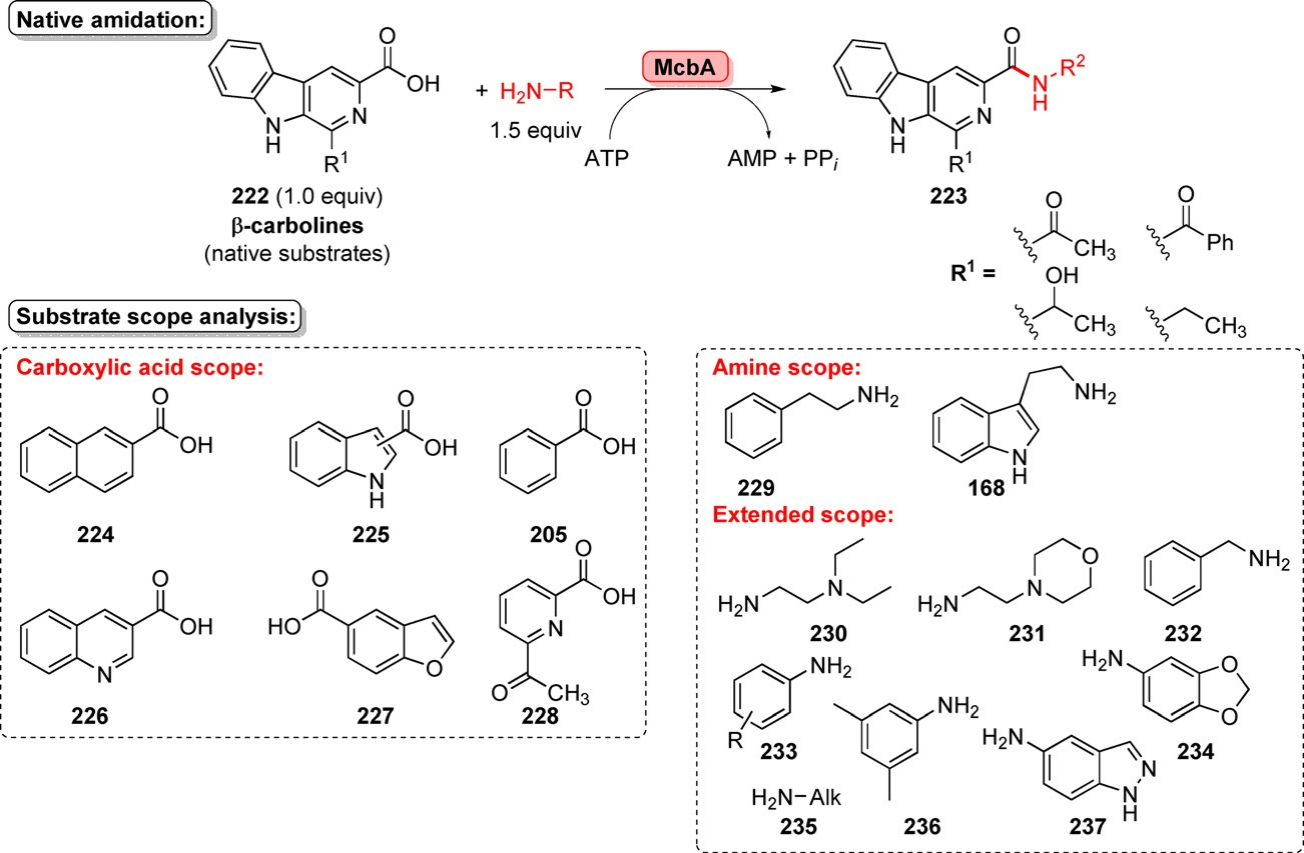
Amide bond synthetase McbA catalyses amidation of β‐carbolines as the native reaction. McbA accepts various aromatic carboxylic acids. The amine scope examined for acid **222** with R^1^=acetyl indicated that diverse aliphatic and aromatic amine building blocks are accepted by McbA.

The synthetic utility of the synthetase McbA from *Marinactinospora thermotolerans* was recently reported by Petchey et al.^[225]^ In the native reaction, McbA catalyses amidation of β‐carbolines (**222**), yet McbA is not exclusively limited to its native substrate (Scheme 38). Broad substrate profiling indicated that a wide range of aromatic carboxylic acids was accepted by McbA.^[226]^ An almost equimolar ratio of acid to amine is a significant advantage towards amide formation, especially on a preparative scale. In further studies, the authors expanded the amine scope towards different aliphatic and cyclic moieties providing a range of interesting building blocks for diversification. Noteworthy was the acylation of poorly nucleophilic amines, particularly shown for a range of anilines (**233**–**237**).^[227]^

Biocatalytic amide bond formation is still in its infancy and investigations into late‐stage diversification have just begun as illustrated by the presented examples. Currently scalability and acid/amine scope as well as the number of available enzymes are the major limitations to be tackled to further evolve this method in the future.

## 6 Reduction of Double Bonds

### 6.1 C=O Reduction

Reduction of C=O double bonds into the corresponding alcohol is an atom economical and alternative approach to stereoselective oxyfunctionalisation. An array of different enzyme families is able to catalyse C=O reductions including ketoreductases (KRED), aldo‐keto reductases (AKRs), medium‐chain dehydrogenases/reductases (MDR), and short‐chain dehydrogenases/reductases that have been thoroughly reviewed by Hollmann et al.^[228]^ In addition to an outstanding significance in industrial biocatalysis, C=O reductions have also emerged in late‐stage synthesis.

**Scheme 39:**
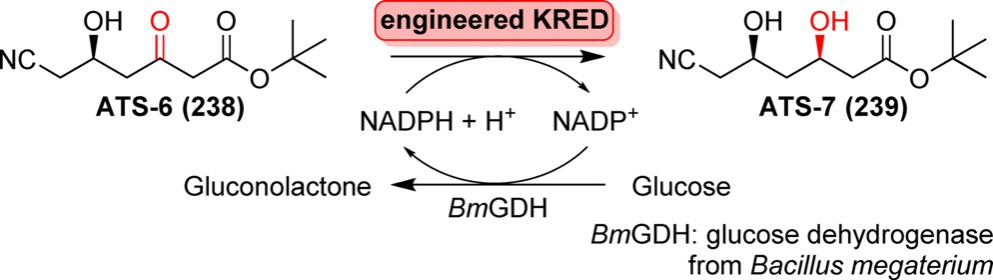
Asymmetric reduction of ATS‐6 by an engineered KRED relevant for the atorvastatin side chain synthesis.

Recently, Gong et al. focused on an LSF approach for the synthesis of atorvastatin, an important cholesterol‐lowering drug.^[229]^ Synthesis of the side chain (ATS‐7, **239**) is an excellent example of a KRED‐catalysed biotransformation due to its high stereoselectivity (Scheme 39). Efforts were undertaken to engineer a KRED variant with increased activity and thermostability by directed evolution affording the chiral alcohol in 87 % yield, >99.5 % *de* on a 100 mL scale at 40 °C.^[229]^

**Scheme 40:**
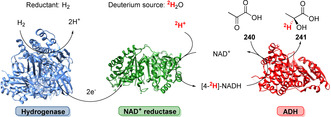
Reductive deuteration of carbonyl groups by making use of a heterogeneous biocatalytic cofactor recycling system along with a KRED and ^2^H_2_O supplying the deuterium atoms.

Interestingly, biocatalytic introduction of deuterium is a notable example of late‐stage labelling by C=O reduction (Scheme 40). The presence of deuterium atoms in pharmaceuticals can result in improved pharmacokinetic properties such as metabolic stability.^[230]^ Rowbotham et al. demonstrated an elegant approach for the asymmetric reductive deuteration by NADH reductases using H_2_ as the reductant along with ^2^H_2_O as an isotope source. Hydrogenase and NAD^+^ reductase were co‐immobilised on carbon particles, enabling the resultant H_2_‐driven system to reduce NAD^+^ to [4‐^2^H]‐NADH. Coupling with a KRED facilitated deuterium transfer onto ketone **240**, thus affording a labelled alcohol (**241**).^[231]^

### 6.2 Late‐Stage Reductive Amination and Amine Oxidation

Biocatalysis has become a powerful tool for C=N reduction to achieve selective amine formation. Several classes of enzymes are capable of performing these reactions, such as imine reductase (IREDs), reductive aminases (RedAms), transaminases, amine dehydrogenases (AmDH), and short‐chain dehydrogenases/reductases (SDRs). Herein, we focus on recent advances of amine synthesis with regard to late‐stage modification.

Biocatalytic reductive amination for the synthesis of pharmaceutical compounds and important chemical building blocks has been reported.[^232^, ^233^] (*R*)‐Rasagiline (**244**) is a drug used for the treatment of Parkinson's disease and is a well‐suited target for RedAms. Briefly, RedAms have the ability to catalyse the asymmetric reductive amination between ketones or aldehydes by combining both imine formation and subsequent reduction (Supporting Scheme S4).^[232]^ In contrast, IREDs require formation of the imine a priori that undergoes subsequent reduction by the enzyme.

**Scheme 41:**
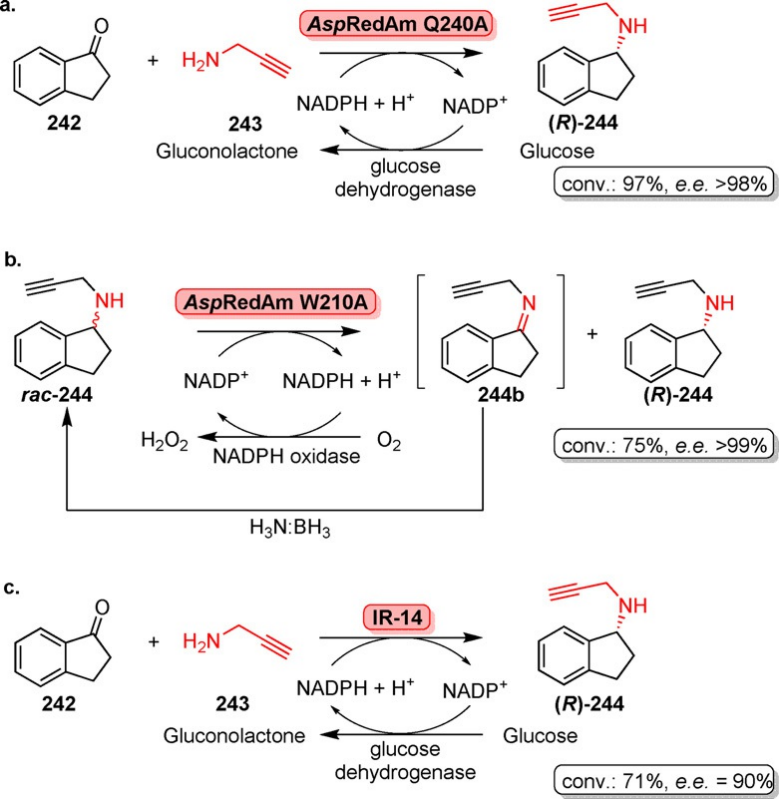
Biocatalytic approaches for (*R*)‐rasagiline (**244**) synthesis.

Synthesis of **244** either started from a prochiral ketone precursor (**242**) or the racemic amine (*rac*‐**244**) by making use of different biocatalytic approaches. Aleku et al. reported on an *Asp*RedAm variant that was able to directly produce **244** starting from **242** and propargylamine (**243**) with >97 % conversion and excellent *ee* (Scheme 41 a).

Another *Asp*RedAm variant with selectivity for the oxidation of (*S*)‐**244** was utilised to exclusively yield imine **244 b**, thus accumulating the desired enantiomer (*R*)‐**244** by dynamic kinetic resolution (Scheme 41 b) with >99 % *ee*.^[234]^ Likewise, Matzel et al. identified an enzyme capable of producing **244** in one step (Scheme 41 c) from the same starting materials.^[233]^

**Scheme 42:**
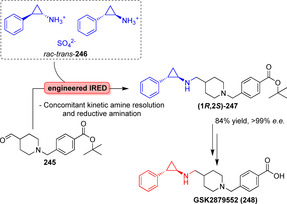
Biocatalytic kinetic resolution and reductive amination towards LSD‐1 inhibitor GSK2879552 (**248**) using a highly engineered IRED.

Manufacture of the lysine‐specific demethylase‐1 inhibitor (GSK2879552, **248**) for the treatment of small cell lung cancer and acute leukaemia provides a recent example of a relevant late‐stage amination:^[235]^ The *tert*‐butyl ester (1*R*,2*S*)‐**247** could be obtained from its late synthetic precursor aldehyde (**245**, Scheme 42). Through extensive engineering preceded by the discovery of a suitable IRED from a previously built panel (IRED‐46)^[236]^ the resultant variant was capable of concomitant kinetic resolution of *rac*‐**246** along with reductive amination. Notably, the engineered enzyme also met the requirements of an industrially viable process.

**Scheme 43:**
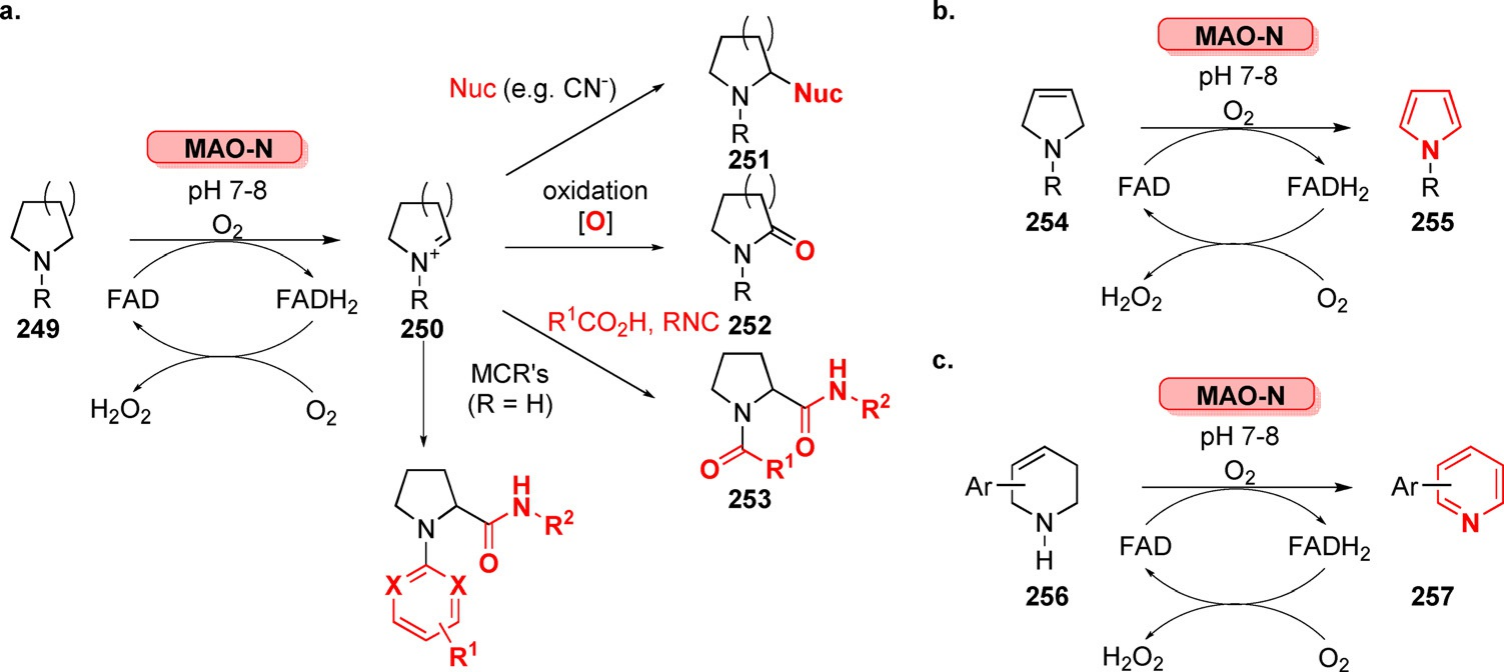
Multiple ways of functionalisation using MAO‐N and its application in heterocycle syntheses. a) Functionalisation α‐ to N‐atom; b) pyrrole synthesis; c) pyridine synthesis.

Amine oxidases can catalyse the opposite reactions to IREDs, opening an attractive tool for multiple types of functionalisations of N‐containing heterocycles. Monoamine oxidase N (MAO‐N) from *Aspergillus niger* has been subjected to extensive rounds of directed evolution and protein engineering resulting in a set of variants (e.g. D5, D9, D10) which have complementary substrate preference coupled with high activity.^[237]^ These variants are particularly active on cyclic five‐membered ring amines and catalyse oxidation to the corresponding imine or iminium under mild conditions (room temperature, pH 7.5, atmospheric dioxygen), a transformation that is equivalent to α‐C−H activation to an amine (Scheme 43). The resulting imines/iminiums have been used for a wide range of transformations including: (i) addition of nucleophiles (e.g. CN, bisulfite); (ii) oxidation to lactams (**252**);^[238]^ and (iii) building blocks for multi‐component reactions, for example, Ugi and Ugi–Smiles.^[239]^ MAO‐N variants have also been used in a different context, to generate both pyrroles and pyridines from appropriate dihydro‐ and tetrahydro‐precursors.[^240^, ^241^] Extensive engineering of MAO‐N proved useful in desymmetrisation of API building blocks leading to enantiomerically pure amine precursors.^[242]^

**Scheme 44:**
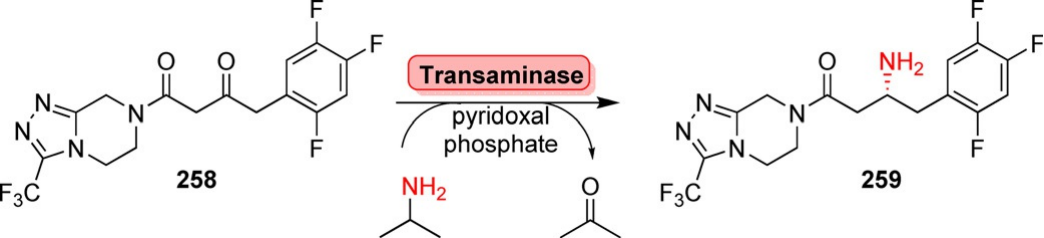
An engineered transaminase facilitates the late‐stage amination of ketone precursor **258** towards sitagliptin (**259**).

The late‐stage amination towards the antidiabetic drug sitagliptin (**259**) represents an outstanding example achieved by Codexis and Merck (Scheme 44).^[243]^ Evolution of a transaminase facilitated reductive amination of the corresponding precursor ketone (**258**) starting from a truncated methyl ketone substrate to shape the active site towards the more demanding ketone which finally gave **259** with excellent enantioselectivity on an industrial scale.

## 7 Emerging Areas of Late‐Stage Modification

### 7.1 Photobiocatalysis

Photobiocatalysis is a novel concept that merges the features of organic photo‐ with biocatalysis which has gained increasing attention in the past decade. In terms of C−H activation, light‐driven reactions enable high catalytic promiscuity along with neat reaction conditions and functional group tolerance.^[244]^ A plethora of photocatalysts, for instance, transition metals or organic frameworks, is well known to utilise light as their energy source whereas development of biocatalytic counterparts is still in its infancy.^[245]^ A comprehensive discussion on the backgrounds of photochemical excitation of enzyme cofactors can be found in a recent review by Sandoval et al.^[246]^

**Scheme 45:**
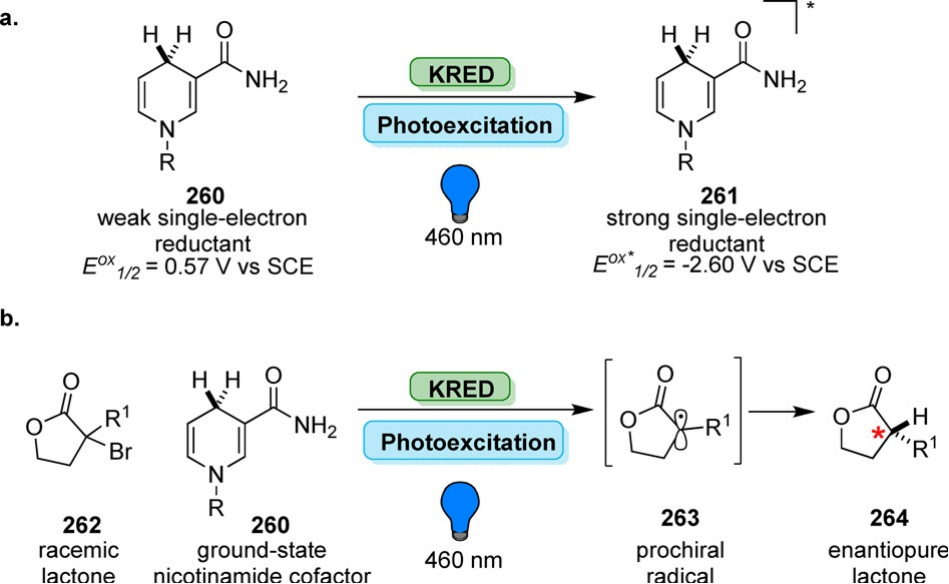
a) Photoexcitation of the NAD(P)H cofactor into a strong single‐electron reductant. b) Application of excited NAD(P)H to the radical dehalogenation of α‐halo lactone **262** to synthesise chiral lactone **264**.

Photocatalytic reactions can be combined or linked with enzyme‐catalysed transformations to unlock new catalytic functions. In particular, NAD(P)H‐dependent enzymes are attractive biocatalytic entities as the cofactor can be excited by visible light. Excitation of the 1,4‐dihydropyridine moiety (**260**) to the strong reductant **261** causes an increase in the reduction potential of NAD(P)H, thereby allowing for reduction of various functional groups when irradiated with blue light (Scheme 45 a).^[247]^

Hyster's group was able to repurpose certain substrate‐permissive enzymes by exploiting photochemical features of their respective cofactors. Excitation of **260** within a KRED was exploited for the dehalogenation of achiral, substituted lactones (**262**). Upon excitation of NAD(P)H, the transformation of a racemic **262** into the dehalogenated enantiopure counterpart **264** succeeded via formation of intermediary radical species **263** (Scheme 45 b).[^247^, ^248^]

In addition to their role in oxyfunctionalisations, P450s have become attractive tools for photobiocatalytic reactions.^[249]^ Previous reports on light‐driven P450‐catalysed reactions include the use of photosystem I (PSI) from plant cells to hydroxylate tyrosine.^[250]^ Tran and co‐workers covalently attached a Ru^II^–diimine complex to the haem domain of P450‐BM3 variants. This hybrid enzyme approach allowed selective hydroxylation of terminal C−H bonds of fatty acids. In addition to reaching high turnover and reaction rates, the photo‐biocatalytic entity omits the need for reductase and NAD(P)H cofactor that streamlines the overall bioprocess compared to native reactions using P450s.^[251]^

**Scheme 46:**
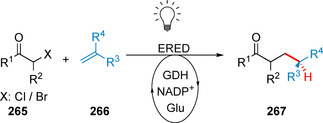
Intermolecular alkylation of terminal alkenes catalysed by EREDs. Adapted from Huang et al.^[252]^

Recently, Huang and co‐workers utilised the light‐driven redox‐catalytic potential of flavin mononucleotide (FMN) by applying it to the enzyme‐catalysed intermolecular alkylation of terminal alkenes (**266**, Scheme 46), thus creating chiral γ‐substituted carbonyl compounds (**267**) found in many bioactive substances such as piperidones, (+)‐3‐oxoabolene, and (*R*)‐4‐methoxyalkanoic acids.^[252]^ Previously characterised substrate‐permissive wild‐type “Ene” reductases (EREDs) were used as biocatalysts so that a variety of α‐halo carbonyl compounds served as substrates. These compounds are able to form an electron donor–acceptor complex with the reduced FMN in the enzyme's active site, which can be excited by visible light to initiate the radical‐based alkylation pathway. Remarkably, excellent yields and enantioselectivities resulted. Further examples can be found in Supporting Section 8 (see Supporting Information).

Although the application of photobiocatalysis in the field of LSF is still in its infancy and further progress must be made to extend the current repertoire, the studies described herein suggest that the ability to re‐task enzymes using light provokes non‐native, highly selective transformations and presents manifold opportunities for the selective functionalisation of multifunctional compounds.

### 7.2 Glycoengineering

**Scheme 47:**
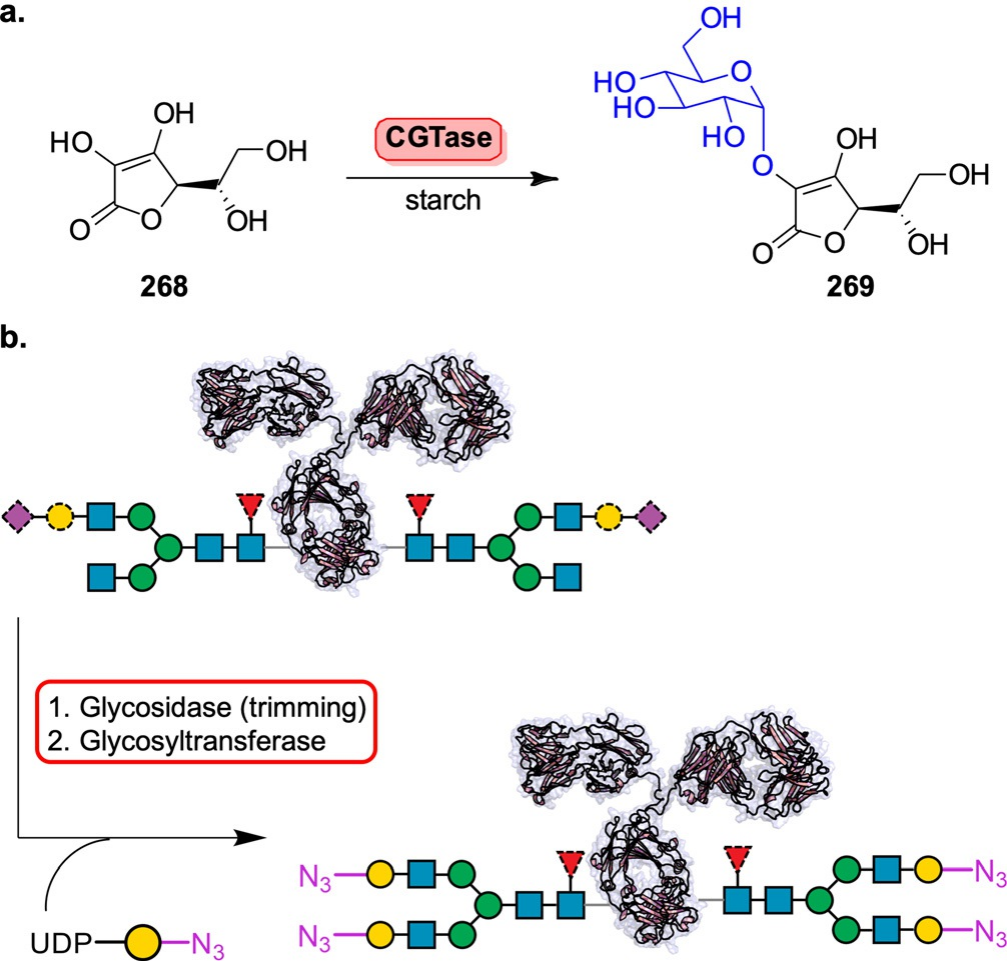
Examples of glycosylation reactions for late‐stage functionalisation. a) Enzymatic glycosylation of small molecules such as ascorbic acid; b) enzymatic glycoengineering of biopharmaceuticals and introduction of non‐natural bioorthogonal groups into the glycan chain of antibodies (glycan nomenclature according to the previously curated standard).^[266]^

Sugars are frequently found attached to small molecules in Nature^[253]^ but are also important parts of many proteins, such as antibodies, and have also been conjugated to DNA.[^254^, ^255^] They have significant impact on biological activity and stability. For example, 2‐*O*‐α‐d‐glucopyranosyl‐l‐ascorbate (**268**) is a bioavailable and stable derivative of ascorbic acid of relevance in the food, beverage, pharmaceutical, and cosmetic industries.^[256]^ The chemical conjugation of glycans to core structures is highly challenging and biocatalysis has been shown to achieve one‐step glycosylation with high selectivity and no need for protection strategies.^[257]^ A typical example is the generation of **268** directly from ascorbic acid using cyclodextrin glycosyltransferase (CGTase), which can catalyse transfer of α‐glucose from starch to a range of alcohols such as ascorbate with control of regioselectivity and retention of configuration at the anomeric centre (Scheme 47 a).^[258]^ Using CGTase for production of chemicals is advantageous to using the more common biosynthetic “Leloir” glycosyltransferases for this process because the latter enzymes require sugar nucleotide substrates which can be recycled using enzyme cascades.^[259]^

Recently, some elegant high‐throughput screening methods have been developed for the discovery of novel glycosylating activities by (meta‐)genome mining from large expressed libraries of glycosidases and glycosynthases.[^260^, ^261^] Remodelling of *N*‐glycans in glycoproteins using biocatalysis has attracted a lot of attention due to extensive applications for biopharmaceuticals such as therapeutic antibodies and antibody–drug conjugates (ADCs). Antibodies generally carry an asparagine‐linked oligosaccharide (*N*‐glycan, Scheme 47 b) that is important for function. The toolbox of biocatalysts that can either trim the *N*‐glycan sequence selectively (endo‐ and exo‐glycosidases) as well as conjugate specific sugars to termini of *N*‐glycan chains (glycosyltransferases) is steadily increasing.^[262]^ Biocatalysis can be used to generate natural glycan structures whereas a number of enzymes also displays promiscuous activity, allowing for introduction of bioorthogonal non‐natural functionalities into glycoproteins. This is of particular interest for the production of ADCs, but also applicable to other proteins.[^263^, ^264^, ^265^]

## 8 Conclusion and Outlook

LSF is an important pillar of modern synthetic organic chemistry where tremendous efforts are being undertaken to develop novel methodologies that are paramount for successful drug development. One‐step transformations on complex scaffolds facilitate diversifications vital to modulate efficacy as well as to improve crucial physicochemical properties, drug metabolism, and pharmacokinetics (DMPK).

Enzymes have recently made their way into late‐stage modifications that are heading in manifold future directions. Already a broad repertoire of biocatalytic transformations can be accessed for obtaining diversified molecular scaffolds. However, high reaction selectivity often means significant biocatalyst specialisation that may result in a restricted substrate profile. Ongoing efforts will solve this downside by (a) protein engineering of niche enzymes, and by (b) (meta‐)genome mining. In addition to a plethora of well‐established examples capable of late‐stage diversification today, many more enzyme‐catalysed transformations will grow to maturity. In light of the remarkable achievements made in biocatalysis over the past decades, the significance of late‐stage biotransformations will undoubtedly expand over the years to come. It can be anticipated that future enzyme catalysis can take a pre‐eminent role in the initial stages of drug discovery, where efforts to miniaturise compound synthesis and screening in a high‐throughput format gain more importance.

There is an almost infinite number of biocatalysts and orthogonal biotransformations utilised in Nature to selectively modify polyfunctional compounds. To date we have only scratched the surface of what is possible.

## Conflict of interest

The authors declare no conflict of interest.

## Biographical Information

*Elvira Romero received her B.Sc. in Biological Sciences from Alcalá University in 2002. She performed her M.Sc. (2005) and Ph.D. (2010) studies at Margarita Salas Center for Biological Research (CSIC), where she studied ligninolytic oxidoreductases. As a postdoc, she investigated enzyme kinetics and mechanisms at Virginia Tech and Georgia State Universities (2010–2014). During the next 4 years at Groningen University, her postdoctoral work was focused on biocatalysis and enzyme engineering. Since March 2019, she is a postdoc in the biocatalysis group at AstraZeneca (Sweden)*.

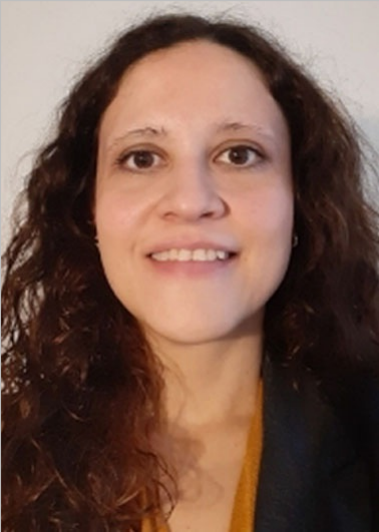

## Biographical Information

*Bethan S. Jones received her MSci in Chemistry with Biomedicine from King's College London in 2018. She is currently undertaking her Ph.D. studies under the supervision of Prof. Sabine L. Flitsch. Her research focuses on oxyfunctionalisation catalysed by cytochrome P450 enzymes, particularly studying applications in early‐ and late‐stage functionalisation within biocatalytic de novo cascades*.

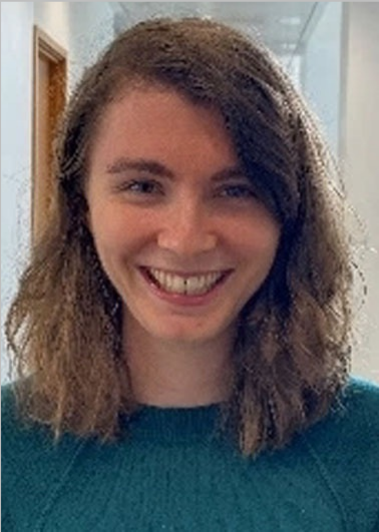

## Biographical Information

*Bethany N. Hogg received her MChem in Chemistry with Medicinal Chemistry in 2019 from the University of Manchester. She has been pursuing her Ph.D. studies since October 2019 under the supervision of Prof. Nicholas J. Turner. Her project is comprised of the production of small molecules through enzyme cascade processes, particularly focussing on the opening of substituted epoxides to afford 1,2‐bifunctional intermediates*.

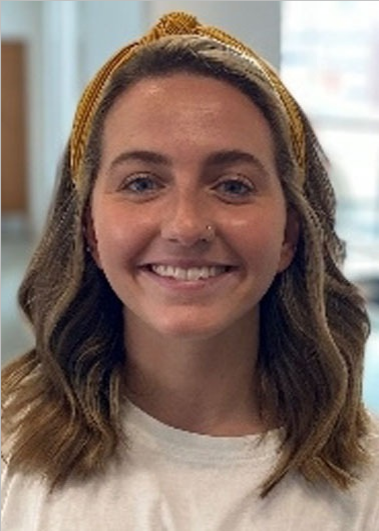

## Biographical Information

*Arnau Rué Casamajo received his M.Sc. degree in Molecular Biotechnology from the University of Barcelona in 2019. He then joined the research group of Prof. Nicholas J. Turner at the Manchester Institute of Biotechnology for his Ph.D. studies. His project focus resides on biocatalytic imine reduction and reductive amination, particularly studying regioselective and enantioselective strategies for the synthesis of pharmaceutically relevant compounds*.

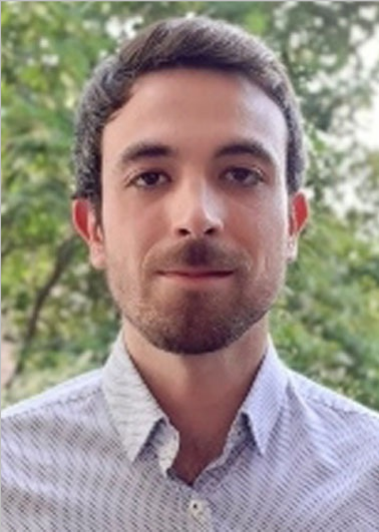

## Biographical Information

*Martin A. Hayes is currently Biocatalysis Leader in the iLAB, Discovery Sciences at AstraZeneca, Gothenburg. He completed his PhD in 1991 with Prof. T. J. Simpson FRS at the University of Bristol. After postdoctoral studies at the University of Toronto with J. Bryan Jones he started his industrial career. He has contributed to the discovery of many small molecule therapeutics including Brilinta and the FLAP inhibitor AZD5718, currently in Phase 2. His research interests include biocatalysis, drug design, biotransformations, and high throughput experimentation*.

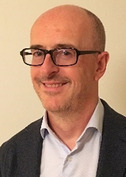

## Biographical Information

*Sabine L. Flitsch is Professor at the School of Chemistry at the University of Manchester. She was educated in Germany, receiving her Diplom in Chemistry from the WWU Münster and then obtained her DPhil under the supervision of Sir Jack Baldwin at Oxford University (UK). After three years as a postdoctoral fellow at MIT (USA) with Professor H. G. Khorana, she moved back to the UK, where she has held academic positions at the Universities of Oxford and Edinburgh, and moved to Manchester in 2005, where she currently holds a Chair in Chemical Biology*.

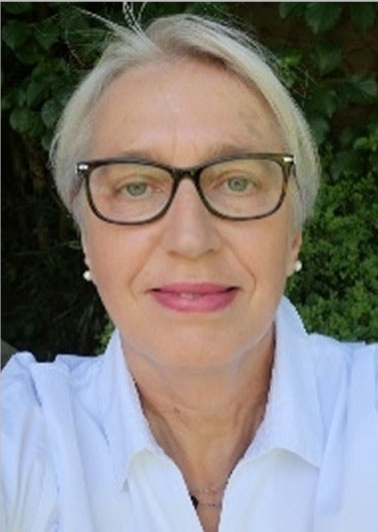

## Biographical Information

*Nicholas J. Turner is Professor of Chemical Biology at the University of Manchester in the Manchester Institute of Biotechnology (MIB: www.mib.ac.uk). He is also Director of the Centre of Excellence in Biocatalysis (CoEBio3; www.coebio3.org). His research interests are in the area of biocatalysis with particular emphasis on the discovery and development of novel enzyme‐catalysed reactions for applications in organic synthesis. His group is also interested in the application of directed evolution technologies for the development of biocatalysts with tailored functions*.

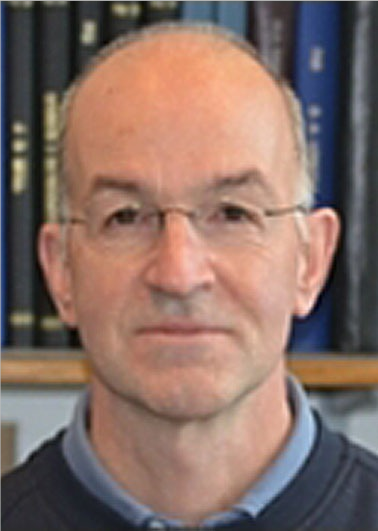

## Biographical Information

*Christian Schnepel received his M.Sc. degree in Biochemistry focusing on Chemical Biology from Bielefeld University (Germany). In 2019, he completed his Ph.D. on biocatalytic halogenation under the supervision of Prof. Norbert Sewald and received the Max‐Bergmann Young Investigator Award. At present, he is a postdoctoral researcher with Prof. Nicholas J. Turner at Manchester Institute of Biotechnology. His research interests include enzymatic synthesis of pharmaceutically relevant targets with special emphasis on methods for amide bond formation and C−H functionalisation*.

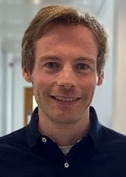

## Supporting information

As a service to our authors and readers, this journal provides supporting information supplied by the authors. Such materials are peer reviewed and may be re‐organized for online delivery, but are not copy‐edited or typeset. Technical support issues arising from supporting information (other than missing files) should be addressed to the authors.

Supplementary
